# Folate Deficiency Triggers the Abnormal Segregation of a Region With Large Cluster of CG-Rich Trinucleotide Repeats on Human Chromosome 2

**DOI:** 10.3389/fgene.2021.695124

**Published:** 2021-07-01

**Authors:** Lorenza Garribba, Ivan Vogel, Mads Lerdrup, Marisa M. Gonçalves Dinis, Liqun Ren, Ying Liu

**Affiliations:** Center for Chromosome Stability and Center for Healthy Aging, Department of Cellular and Molecular Medicine, University of Copenhagen, Copenhagen, Denmark

**Keywords:** aneuploidy, fluorodeoxyuridine, fragile site, FRAXA, nondisjunction, MiDAS

## Abstract

Folate deficiency is associated with a broad range of human disorders, including anemia, fetal neural tube defects, age-associated dementia and several types of cancer. It is well established that a subgroup of rare fragile sites (RFSs) containing expanded CGG trinucleotide repeat (TNR) sequences display instability when cells are deprived of folate. However, given that folate sensitive RFSs exist in a very small percentage of the population, they are unlikely to be the cause of the widespread health problems associated with folate deficiency. We hypothesized that folate deficiency could specifically affect DNA replication at regions containing CG-rich repeat sequences. For this, we identified a region on human chromosome 2 (Chr2) comprising more than 300 CG-rich TNRs (termed “FOLD1”) by examining the human genome database. Via the analysis of chromosome shape and segregation in mitosis, we demonstrate that, when human cells are cultured under folate stress conditions, Chr2 is prone to display a “kink” or “bending” at FOLD1 in metaphase and nondisjunction in anaphase. Furthermore, long-term folate deprivation causes Chr2 aneuploidy. Our results provide new evidence on the abnormalities folate deficiency could cause in human cells. This could facilitate future studies on the deleterious health conditions associated with folate deficiency.

## Introduction

Folate is a B vitamin that functions as a carrier for one-carbon units that are essential for DNA synthesis (Ducker and Rabinowitz, 2017). Humans cannot synthetize folate and therefore rely on a dietary supply of this nutrient. In human populations where folic acid supplementation is absent, folate deficiency is frequently observed (Bailey et al., 1982; Senti and Pilch, 1985; Roman Vinas et al., 2011; Mensink et al., 2013). This deficiency is known to be associated with anemia (Green and Miller, 1999), fetal neural tube defects (No authors listed, 1991), infertility in men and women (Altmae et al., 2010; Gaskins et al., 2012; Sansone et al., 2018), age-associated dementia, psychological disorders (Araujo et al., 2015), and several types of cancer (including breast, pancreatic, colon, esophageal, and gastric) (Giovannucci, 2002; Larsson et al., 2006; Ericson et al., 2007). Notably, it is established that a subgroup of so-called rare fragile sites (RFSs), which contain CGG trinucleotide repeat (TNR) sequences (Durkin and Glover, 2007), are particularly sensitive to the intracellular level of folate. Among them, FRAXA is the most widely studied RFS and is associated with fragile X syndrome (FXS) when the TNR sequence expands beyond a critical length (Pieretti et al., 1991; Santoro et al., 2012). RFSs appear on metaphase chromosomes as gaps or breaks in otherwise fully condensed chromatin (called RFS “expression”). However, because they are present in a very small subset of individuals (less than 5%), RFSs containing abnormally expanded CGG-TNR sequences are unlikely to be the cause of the more widespread health problems associated with folate deficiency.

It is known that folate deficiency has a negative impact on genome stability. Previous studies showed that cells deprived of folate exhibit DNA replication-associated DNA breakage (Lamm et al., 2015) and chromosome instability (e.g., chromosome 8 aneuploidy) (Ni et al., 2010). In agreement with this, our previous work demonstrated that, in response to folate stress, the FRAXA locus is dramatically missegregated in mitosis (Bjerregaard et al., 2018). Importantly, long-term folate deprivation not only exacerbates FRAXA instability but also leads to chromosome X aneuploidy (Bjerregaard et al., 2018). However, it remains unknown whether other “non-RFS” regions are also susceptible to folate deprivation. Considering that all of the 24 folate sensitive RFSs contain abnormally long CGG-TNRs (Durkin and Glover, 2007), we hypothesized that folate deficiency might specifically affect genomic regions containing apparently non-pathological CG-rich repeat sequences. It is conceivable that folate deficiency could specifically interfere with the replication of these CG-rich sequences, which potentially can form DNA secondary structures due to the slowing of replication forks in response to a lack of thymidine and/or purines (Fry and Loeb, 1994; Usdin and Woodford, 1995). The resulting replication perturbation would then interfere with the timely locus condensation in early mitosis and the subsequent sister chromatid disjunction in anaphase, as is observed at FRAXA (Bjerregaard et al., 2018). This, in turn, would lead to heritable alterations at CG-rich TNR regions, and potentially explain the diverse health problems associated with folate deficiency.

To address this hypothesis, we conducted a bioinformatic analysis of human genome and identified a region at 2p11.2 that contains a cluster of more than 300 CG-rich TNRs (termed FOLD1 for “folate-deficiency-induced bending 1”). Our results demonstrated that folate deprivation leads to distinct mitotic abnormalities at FOLD1, possibly due to a difficulty in replicating the CG-rich TNRs in this region when cells are subjected to folate stress.

## Materials and Methods

### Cell Lines

Epstein-Barr virus immortalized human B-lymphocyte GM09237 (male, with 931–940 CGG repeats (Bjerregaard et al., 2018) at the *FRAXA* locus) was from the Coriell Biorepository. Untransformed foreskin fibroblast Hs68 cells and osteosarcoma cell line U2OS were from the ATCC.

### Cell Culture

B-lymphocytes were maintained in RPMI 1640 Medium (Gibco), while Hs68 cells were maintained in Dulbecco’s modified Eagle’s medium (DMEM). In both cases, the medium was supplemented with 15% fetal bovine serum (FBS, Thermo Fisher Scientific). U2OS cells were maintained in DMEM supplemented with 10% FBS. The cells were maintained at 37°C in a humidified atmosphere with 5% CO_2_ and were routinely subjected to mycoplasma testing (using MycoAlert; Lonza). Only mycoplasma-free cells were analyzed.

### Cell Synchronization and Treatment

To obtain metaphase chromosomes, asynchronous cells were cultured under several different conditions including untreated (Unt), with 0.5 μM 5-fluorodeoxyuridine (FdU; Abcam), 0.4 μM aphidicolin (APH; Sigma Aldrich) or 0.4 mM hydroxyurea (HU; Sigma Aldrich) for 22 h and were synchronized in metaphase by being cultured with colcemid (100 ng/ml; Thermo Fisher Scientific) for the last 5 h of the 22-h treatment. For “No folate” condition, cells were cultured in RPMI 1640 medium without folic acid (Gibco), or DMEM medium without folic acid (Cell Culture Technologies) for 3 or 5 days and were then synchronized at metaphase by being cultured with colcemid (100 ng/ml; Thermo Fisher Scientific) for the last 5 h before harvesting.

To analyze mitotic DNA synthesis (MiDAS) on metaphase chromosomes, asynchronous cells under untreated or FdU treated conditions were arrested in late G2 using 12 μM RO3306 (12 μM; Merck) for 9 h. Cells were then released into medium containing 20 μM 5-ethynyl-2′-deoxyuridine (EdU; A10044 Thermo Fisher Scientific) and colcemid (100 ng/ml; Thermo Fisher Scientific) and were then incubated for 1 h before harvesting.

To harvest anaphase/telophase cells, asynchronous cultures were treated with RO3306 for 9 h to induce an arrest at the G2/M boundary. Cells were then released from RO3306, incubated for 30 or 45 min in pre-warmed cell culture medium (37°C), and then seeded onto poly L-Lysine coated slides (Sigma Aldrich) prior to fixation. For cells cultured in RPMI 1640 without folic acid (“No folate”), RO3306 was added to the medium after 3- or 5-day incubation, and the cells were then released after 9 h as indicated above.

To assess chromosome nondisjunction, a previously published protocol was used (Bjerregaard et al., 2018). Briefly, cells were released from FdU into medium containing 10 μM EdU (Thermo Fisher Scientific) for 3 h to label the cells that had gone through S phase in the presence of FdU. Cells were then released into cytochalasin B (3 μg/ml; Sigma-Aldrich) for 16 h and seeded onto poly L-Lysine slides (Sigma-Aldrich) for further analysis.

### Fluorescence *in situ* Hybridization

To analyze metaphase chromosomes, cells in metaphase were harvested using standard procedures. Briefly, cells were swollen in 75 mM KCl (at 37°C), fixed in methanol:acetic acid (3:1) and then dropped onto glass slides. To analyze anaphase and cytokinesis-blocked cells, cells were seeded on poly-L-Lysine slides (Sigma-Aldrich) and fixed in methanol:acetic acid (3:1) for fluorescence *in situ* hybridization (FISH) analysis. BAC clones were used to prepare FISH probes targeting the regions of interests: RP11-439L14 (GenBank: AC012454.8) for FOLD1, RP11-464P18 (GenBank: AC073105.1) for Chr2Q, RP11-799C6 (GenBank: AC068519.1) for Chr1CCEN, RP11-383P16 (GenBank: AC233288.1) for FRAXA (Supplementary Figure 1A). Probes were labeled using the BioNick labeling system (Thermo Fisher Scientific) or DIG-nick translation mix (Sigma Aldrich). FISH was carried out using standard procedures, as previously published (Bjerregaard et al., 2018). Slides were mounted using Vectashield mounting medium with DAPI (Vector Labs). Images were captured using an Olympus BX63 microscope. Images were analyzed using CellSens (Olympus) or Fiji/ImageJ software. In the analysis of “bent” shape of human chromosome 2 (Chr2), all of the images were analyzed independently by two researchers.

### Karyotype Analysis

Karyotypes of metaphase cells were obtained using conventional G-banding and were analyzed using a Leica light microscope equipped with CytoVision software.

### FISH/EdU Detection

To detect EdU incorporation at FOLD1, revealed by FISH, we followed a previously described protocol (Garribba et al., 2018) with minor modifications. Briefly, at the end of the FISH procedure, slides were fixed in 4% paraformaldehyde (PFA) in PBS for 4 min at room temperature (RT) and then blocked in 3% bovine serum albumin (BSA) in PBS + 0.5% Triton-X100 for 10 min at RT. Then, EdU detection was carried out using Click-iT^®^ Plus EdU Alexa Fluor^®^ 594 Imaging Kit (C10339 Thermo Fisher Scientific). Slides were incubated with the reaction mix for 1 h at RT in the dark and were then washed in 3% BSA in PBS + 0.5% Triton-X100 (3 × 20 min). Slides were mounted using Vectashield mounting medium with DAPI (Vector Labs). Images were captured using an Olympus BX63 microscope and analyzed using CellSens (Olympus) or Fiji/ImageJ software.

### Statistical Analysis

At least three independent experiments were carried out to generate each data set. Statistical significance in each case was calculated using Chi-squared or Fisher’s exact test for all of the data sets.

### Genome Sequencing and Bioinformatic Analysis

#### Extraction of Simple Repeats

AT or CG rich simple repeats were obtained from the recent version of the human genome assembly (GRCh38/hg38) curated at UCSC Genome Browser Database (Rosenbloom et al., 2015). The number of nucleotides of repeat unit ranges from 1 to 14. Considering that the average GC-content in human genomes ranges from 35 to 60%, with a mean of 41% (Piovesan et al., 2019), we reasoned that, if a region has more than 60% C/G repeats, then it will have more C/G than the other parts of the genome. Therefore, we used 60% as a threshold to filter out the regions with simple repeats. Three databases, namely “RepeatMasker Database,” “Tandem Repeat Finder,” and “Microsatellites,” were used to extract repeats. To identify TNRs, the RepeatMasker database was used, while the repeats were concatenated and calculated with the other two databases. To extract and organize potential repeat sequences, genomic features including chromosome position, length of the repeat unit, number of repetitions of the repeat unit, total span of the repeat chain, the overall similarity of the consensus sequence to the repeat chain, consensus sequence, and database source were included (Supplementary Tables 1, 2). The extraction process was performed using an automated computational pipeline that includes custom-made Bash and Python scripts. The scripts can be accessed at github: https://github.com/puko818/trinucl-hunter.

#### Overlap Analysis With Genomic Features

To analyze the overlap between repeat sequences and genomic features, we obtained BED files from the following databases (Quinlan and Hall, 2010): (1) CpG islands (database table cpgIslandExt) (hg38); (2) Cytogenetic bands (database table cytoBand) (hg38); (3) Promoters (database table knownGene, with positions 2000 bases upstream and 500 bases downstream of the transcription start site) (hg38); (4) Exons (database table knownGene) (only positions of the exons from the downloaded coordinates (hg38); (5) introns (database table knownGene) (only positions of the introns from the downloaded coordinates) (hg38); (6) transposable elements (TEs; database table rmsk) Records labeled as simple repeats were skipped as those were already included in the AT/CG-rich database (hg38); (7) transcription factor binding sites (TFBS; database table wgEncodeRegTfbsClusteredV3) (hg19) (with coordinates being converted to h38 coordinates using the liftOver tool) (Rosenbloom et al., 2015); (8) COSMIC database of somatic mutations – database table COSMIC (hg19) (with coordinates being converted to hg38 using the liftOver tool; (9) polymorphisms (database of single nucleotide polymorphisms-database table snp147Common from hg38 and database of genomic variants - database table dgvMerged from hg38). BEDOPS package (Neph et al., 2012) was used to analyze the overlaps. The criterion for designation of an overlap was 1 bp. The expected or observed representation of the repeats in certain genomic features was calculated using the following formula:

$$\mathrm{Expected}\,\mathrm{representation}:\frac{\sum_{i=1}^{n}\left|r\,e\,p\,e\,a\,t_{i}\right|.\sum_{j=1}^{m}\left|f\,e\,a\,t\,u\,r\,e_{j}\right|}{{\left|g\,e\,n\,o\,m\,e\right|}^{2}}$$

$$\mathrm{Observed}\,\mathrm{representation}:\frac{\sum_{j=1}^{m}\sum_{i=1}^{n}\left|r\,e\,p\,e\,a\,t_{i}\cup f\,e\,a\,t\,u\,r\,e_{j}\right|}{\left|g\,e\,n\,o\,m\,e\right|}$$

|*repeat*_*i*_|is the length of the i-th repeat from the group of AT-rich or CG-rich repeat database of size *n*, |*feature*_*j*_|is the length of the j-th feature from the particular database of genomic features of size m. In detail, feature span corresponds to $\sum_{j=1}^{m}\left|f\,e\,a\,t\,u\,r\,e_{j}\right|$ and repeat span corresponds to $\sum_{j=1}^{n}\left|r\,e\,p\,e\,a\,t_{j}\right|$. These are both normalized to the length of the genome (|*genome*|^2^ in the denominator). Repeatspan refers to the total lengths for all such repeats within the genome. Featurespan is the total length for all such features across the genome. Nonrandom association of specific features with repeat categories was assessed based on observed association compared to an expectation of random distribution. Subsequently the expected and observed representations of overlapped genomic features with repeats were analyzed using ggplot2 and displayed as Log fold changes.

#### Genome Sequencing and Data Analysis

##### Secondary generation of whole-genome sequencing

Two DNA samples extracted from GM09237 cell line cultured with either normal medium (untreated; labeled as “YL2.1.18”) or medium with no folic acid for 5 days (no-folate-5-day; labeled as “YL2.2.18.mixed”) were sequenced by BGI PE100 platform (BGI whole genome 100 bp paired-end sequencing 60×; BGISEQ PE100). These two whole-genome sequencing (WGS) data can be accessed at: https://ega-archive.org/datasets/EGAD00001007732. WGS data quality was assessed using FastQC (available online at: http://www.bioinformatics.babraham.ac.uk/projects/fastqc/ (2015), “FastQC,” https://qubeshub.org/resources/fastqc), MultiQC (Ewels et al., 2016) and Fastq Screen (Wingett and Andrews, 2018). New WGS data were trimmed using “fastp” version 0.20.0 (Chen et al., 2018) with the settings of “detect_adapter_for_pe –correction –trim_poly_x –cut_tail –trim_front1 2 –trim_front2 2,” and mapped using “bowtie 2” (version 2.2.9) (Langmead and Salzberg, 2012) with the setting of “local,” and converted to bam files using Samtools (version 1.10) (Li et al., 2009) with the settings of “view – Sb.” The WGS data for the U2OS cell line was obtained from Akan et al. (2012) as bam files and assembled accordingly.

##### Evaluation of CG content in the genome

While optimizing the mapping at the hard to map regions including FOLD1 and FRAXA, we tested a range of different mapping settings including “–k 2” or “–k10000” to allow more multimapping regions in the output as well as “bwa mem” (Li, 2013). In addition, we generated fastq files consisting of 100 bp pseudoreads based on the reference genome sequence of the regions and their surroundings. While the pseudoreads could be mapped with high success-rates using the standard pipeline described above, none of the more permissive settings resulted in mapped reads within the regions. We therefore concluded that the lack of reads mapped to FOLD1 is not due to repetitive sequences or low “mappability” in general, but likely due to the exceptionally high GC content of the locus (92.6%), which may inhibit its PCR amplification, cluster formation in the flow cell and/or the sequencing reaction. Indeed, under standard library preparation procedures, the abundance of regions with a GC content >65% are reduced about hundred times compared to mid-GC reference regions after just ten PCR cycles (Aird et al., 2011).

To validate this in detail, FASTA files of hg38 genome sequence were downloaded from UCSC (Karolchik et al., 2004) and used to derive a bedgraph file with the C/G percentage in 100 bp in windows, as well as bed files with coordinates for each CpG dinucleotide or GCC/GGC trinucleotide. These files and bam files with mapped sequencing reads were imported as “datasets” in EaSeq (Lerdrup et al., 2016) using default settings, except for WGS data, where the option to filter for unique reads was disabled. A list of chromosome lengths in hg38 was imported into EaSeq as a Regionset and used to generate 1,000 bp windows covering the entire reference genome using the “Modify”-tool in the Regionset-panel with the setting “Homogenize and fragment regions to 1,000 bp.” The number of CpG and GCC/GGC coordinates as well as average C/G-percentage in each window was obtained for this set of regions using the “Quantify”-tool with all normalization options disabled. Genome-wide data from this set of regions were visualized using the “Scatter” plot type in EaSeq, with scales adjusted as shown in the plots and 100 bins on each axis. Tracks were visualized using the “FillTrack” plot type with *Y*-axis settings adjusted as indicated in the figures and with normalization and smoothing disabled. Plots were exported as pdfs from EaSeq and lay-outed in Adobe Illustrator CS6.

##### Third generation of WGS

Two DNA samples extracted from GM09237 cell line cultured with either normal medium (untreated; labeled as “YL2.1.18”) or medium with no folic acid for 5 days (no-folate-5-day; labeled as “YL2.2.18.mixed”) were sequenced by PacBio Sequel system (SMARTbell; performed by BGI). Raw data of this sequencing can be accessed at: https://ega-archive.org/datasets/EGAD00001007732. Long reads were mapped using two different algorithms: Minimap2 version 2.17 with the settings -ax map-pb (Li, 2018) and NGMLR version 0.2.7 (Sedlazeck et al., 2018). Reads which mapped using either algorithm were further excised from SAM output using awk and realigned for visualization using BLAST and Sequencher (Gene Codes Corporation, United States).

## Results

### CG-Rich TNRs Are Frequently Associated With “Functional Genomic Regions”

**FIGURE 1:**
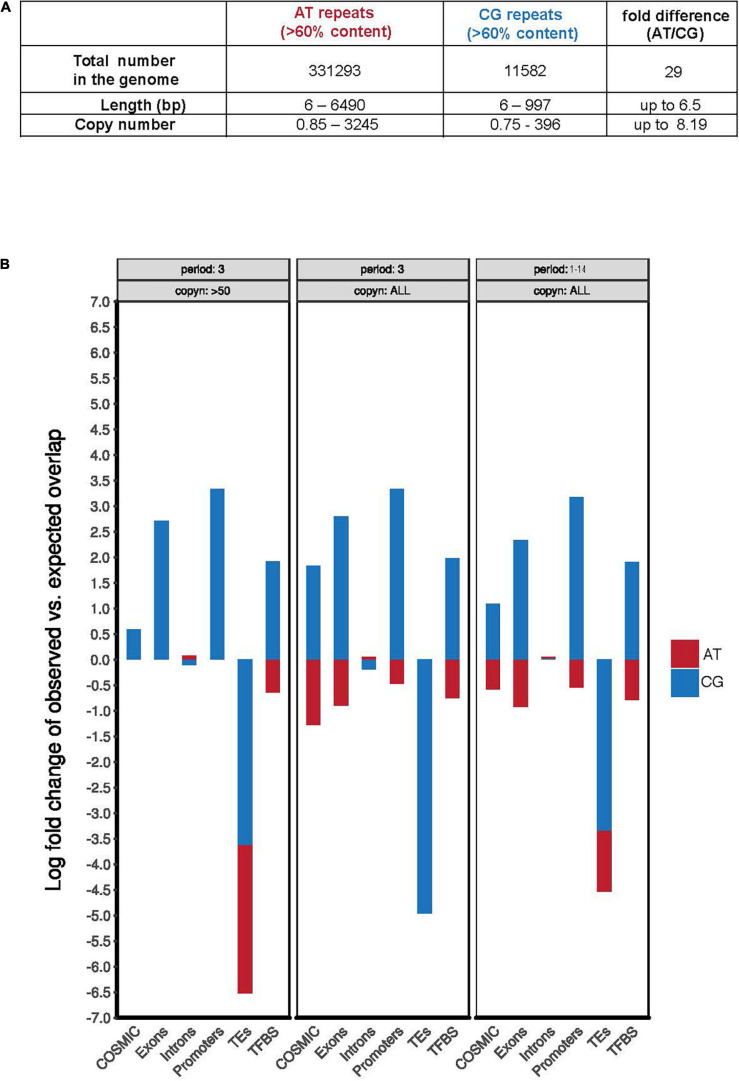
CG-rich TNRs are frequently located at “functional genomic regions.” **(A)** Summary of the total number, variation of length, and copy number of AT and CG repeats in human genome derived from Supplementary Table 1. **(B)** Quantification of the observed AT-rich (red) or CG-rich (blue) repeats in selected genomic features using theoretically predicted distribution frequency as a baseline. The quantification was performed respectively for repeats of period of 3 bp and with more than 50 copies (left), repeats of period of 3 bp (middle), or repeats of period length ranging from 1 to 14 bp (right). COSMIC is the Catalogue of Somatic Mutations in Cancer database. Copyn: copy numbers. TEs: transposable elements. TFBS: transcription factor binding sites.

To examine whether folate deprivation might adversely affect CG-rich regions of the human genome that have not been implicated as disease-causing, we performed a bioinformatic analysis of the GRCh38/hg38 human genome assembly^1^ to identify regions characterized by the presence of extended AT-rich or CG-rich simple repeats. In agreement with earlier work on the composition of the human genome (Burge et al., 1992; Nakashima et al., 1997; Lander et al., 2001; Venter et al., 2001), our analyses illustrated two marked differences between AT-rich and CG-rich repeats: (1) the number of AT-rich repeats in the human genome far exceeds that of CG-rich repeats (nearly 30-fold higher); (2) AT-rich repeats display a much wider range of repeat sequence classes and copy numbers than that of the CG-rich repeats (Supplementary Table 1 and Figure 1A). This relatively lowered abundance and diversity of CG-rich repeats likely reflects the deamination of 5-methylcytosine during evolution (Tomso and Bell, 2003; Fryxell and Moon, 2005; Walsh and Xu, 2006). When focusing on the TNR sequences we, however, found that the copy numbers of AT-rich or CG-rich repeats was very similar, ranging from 1 to 161 or 1 to 332, respectively (Supplementary Table 1). This suggested that CG-rich TNRs have been retained during evolution. Possibly, this reflects codons or the local aggregation of CpG-dinucleotides in thousands of CpG-islands, which are characterized by the overall low cytosine methylation levels, and the consequent protection from 5-methylcytosine deamination (Gardiner-Garden and Frommer, 1987; Antequera et al., 1989; Deaton and Bird, 2011; Long et al., 2016). In view of this, we compared the frequency of AT- or CG-rich repeats located in functional genomic regions, e.g., exons, promoters, TEs, TFBS, and regions mutated in cancer (COSMIC) (Figure 1B). This analysis showed that all of the CG-rich simple repeats, including TNRs, are more significantly enriched in functional regions including exons, promoters, TFBS, and regions mutated in cancer (Figure 1B and Supplementary Tables 1, 2). This is consistent with the notion that GC-rich sequences are preferentially retained in gene-rich and highly transcribed regions in the genome of warm-blood animals due to its thermostability, which helps in maintaining the advanced level of genomic organization in these animals (the “transcription/grade” concept) (Vinogradov, 2003). Taken together, this analysis indicates that CG-rich TNRs have a similar copy number range to that of AT-rich TNRs, and are enriched in functional genomic regions in the human genome.

### Folate Deficiency Causes “Bending” at Chr2p11.2, FOLD1

**FIGURE 2:**
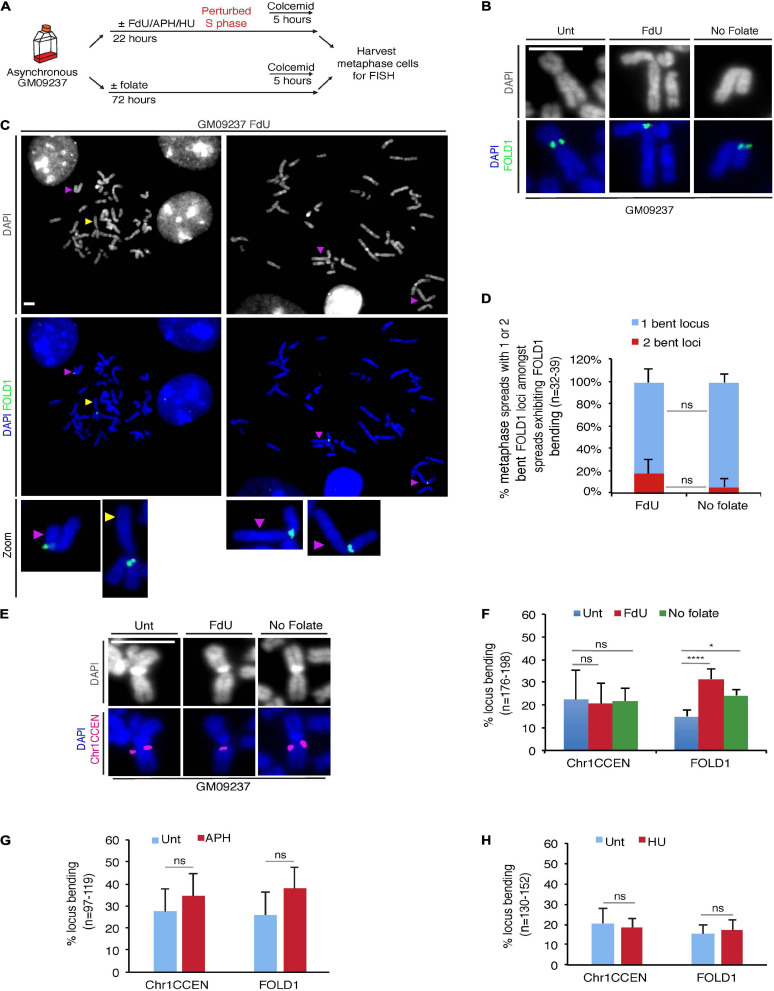
Chr2 exhibits a “kink” at FOLD1 following FdU treatment or folate deprivation. **(A)** Experimental workflow for the analysis of metaphase cells following FdU treatment for 22 h (FdU) or folate deprivation for 3 days (No folate) in GM09237 cells. **(B)** Representative images of kinked Chr2 at FOLD1 (green) in GM09237 cells. **(C)** Representative images and **(D)** quantifications of metaphase spreads with either 1 or 2 bent Chr2 at FOLD1 (green) in cells exhibiting Chr2 bending. An “un-bent” Chr2 is indicated by a yellow arrowhead, and “bent” Chr2s are indicated by purple arrow/heads. Selected chromosomes are shown as zoomed images below. *N*: number of metaphase spreads analyzed. **(E)** Representative images of Chr1 at Chr1CCEN. The location of Chr1CCEN (magenta) is revealed by FISH analysis. **(F)** quantifications of kinked Chr2 at FOLD1 or Chr1 at Chr1CCEN in GM09237 cells. *N*: number of Chr2s or Chr1s analyzed. **(G,H)** Quantification of kinked Chr2 at FOLD1 or Chr1CCEN under aphidicolin (APH) or hydroxyurea (HU) in GM09237 cells. *N*: number of Chr2s or Chr1s analyzed. Scale bar, 5 μm. Data are means of at least three independent experiments. Error bars represent SD (ns, not significant; *p < 0.05; ****p < 0.0001).

Amongst the CG-rich TNR sequences, we identified a region at Chr2p11.2, that we termed FOLD1, which contains the largest cluster of CG-rich TNRs (∼333) in the human genome (Supplementary Table 1). To test our hypothesis that folate deficiency could affect the stability of regions containing large number of repeats, we started our analysis with a FXS mutant cell line GM09237, which is known to contain more than 900 CGG repeats at the FRAXA locus (Vincent et al., 1991; Seneca et al., 2012) and display missegregation in mitosis when it is cultured under folate stress conditions (Bjerregaard et al., 2018). First of all, we confirmed that either of the two types of widely-used folate stress-inducing treatments could induce fragility at FRAXA. For this, we treated the cells with FdU (a thymidylate synthase inhibitor that disrupts thymidine production (Wilson et al., 2014)) for 22 h, or deprived the cells of folate for 3 days (Bjerregaard et al., 2018), and then harvested metaphase cells for FISH using DNA probes targeting the FRAXA locus (Supplementary Figure 1). We then performed the same analysis with a DNA probe targeting the FOLD1 region (Supplementary Figure 1A and Supplementary Text 1). Our results showed that, although FOLD1 region did not exhibit a gap or break as observed at fragile sites, there was a recognizable structural abnormality at this locus in response to folate stress, which took the form of a “bending” or “kink” centered at FOLD1 with an angle less than 90°. We have subsequently quantified this bending shape of Chr2 in metaphase spreads obtained from different samples. Our results indicated that this unusual bending of Chr2 is consistently associated with folate stress (Figures 2A,B,F). In addition, we could observe that, amongst the metaphase spreads exhibiting Chr2 bending, a proportion of them have both copies of Chr2 showing distinct bending (19% versus 6% in “FdU” and “No-folate” conditions, respectively; Figures 2C,D). This suggests that the bending of Chr2 is not exclusive to either the maternal or the paternal copy of Chr2. Given that bending occurs on approximately 30% of the copies of Chr2, it would be expected statistically that, in average, 9% of the cells would show bending on both copies of Chr2, as was observed.

To exclude the possibility that this phenotype was due to the large size of Chr2, we studied a region on the largest human chromosome, Chr1. This region is located at Chr1p12-21 (henceforth referred as Chr1CCEN) and lies proximal to the centromere on the shorter arm of Chr1 at a very similar position to that of FOLD1 on Chr2 (Supplementary Figure 1A). However, Chr1CCEN differs from FOLD1 in lacking any long CG-rich TNRs. We observed that, unlike FOLD1, the Chr1CCEN region was not associated with any consistent structural abnormality in response to folate deprivation or FdU treatment, suggesting that specific forms of chromosome “bending” are not a universal feature of large chromosomes *per se* (Figures 2E,F). Furthermore, we could confirm that Chr2 bending at FOLD1 was not induced by APH or HU (Figures 2G,H), which are known to perturb replication and induce common fragile site (CFS) (Glover et al., 1984) or early replicating fragile site (Barlow et al., 2013) expression, respectively.

We subsequently performed the same analysis on two more cell lines: a non-cancerous fibroblast cell line (Hs68) and an osteosarcoma cell line (U2OS). Our results indicated that, in these two cell lines, under folate stress conditions, Chr2 is also prone to bend at FOLD1, while Chr1 does not show increased bending at Chr1CCEN, which is similar to what we observed in the GM09237 cell line (Supplementary Figures 2A,B). We have also carried out conventional karyotyping to study the shape of Chr2 and Chr1 in the GM09237 and Hs68 cell lines, both of which are non-cancerous and diploid. The data from this analysis indicated that Chr2 indeed has a more pronounced bending shape when the cells are cultured under folate stress conditions, which is not the case for Chr1s or Chr3s, the two other largest human chromosomes (Supplementary Figures 2C,D).

Next, we asked whether MiDAS, which serves as a rescue pathway to complete locus duplication in mitosis, and is known to occur at CFSs (Minocherhomji et al., 2015), telomeres (Minocherhomji et al., 2015; Dilley et al., 2016; Min et al., 2017; Ozer et al., 2018) and FRAXA (Garribba et al., 2020), could occur at FOLD1 following folate stress treatment. To address this, we investigated whether mitotic EdU incorporation could be observed at the sites of bending FOLD1 following folate stress using an established protocol (Garribba et al., 2018) in GM09237 cells (Supplementary Figure 3). Although we could indeed see MiDAS at FRAXA as we observed previously, we could not detect MiDAS at FOLD1 after examining more than 150 metaphase spreads obtained from three replicate experiments. This suggests that, unlike FRAXA, the FOLD1 region is most likely fully replicated before mitosis.

### FOLD1 Is Missegregated Under Folate Stress Conditions

**FIGURE 3:**
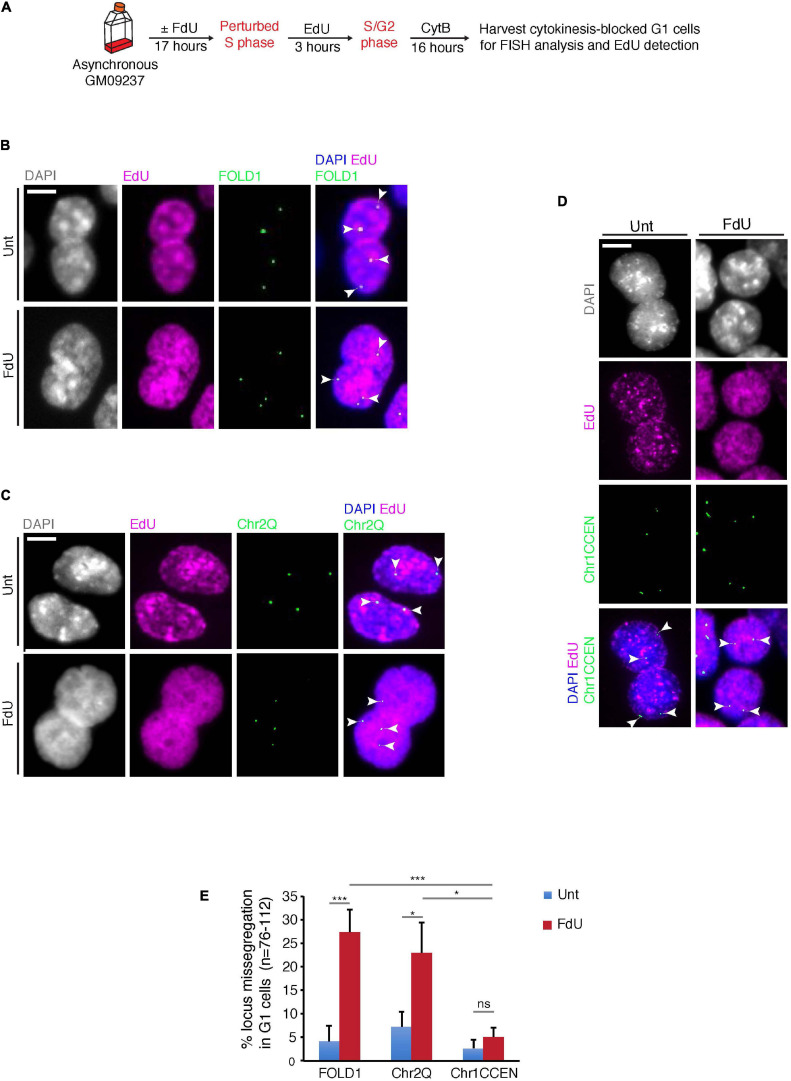
FOLD1 displays frequent nondisjunction in G1 cells following FdU treatment. **(A)** Experimental workflow for quantifying FOLD1 nondisjunction in newly-born G1 cells following FdU treatment for 17 h in GM09237. **(B–D)** Representative images and **(E)** quantification of cytokinesis-blocked binucleated (G1) cells following FdU treatment of GM09237 cells. White arrowheads in panels **(B–D)** denote the location of FISH probes. *N*: number of pairs of newly-born G1 cells analyzed. Scale bar, 5 μm. Data are means of at least three independent experiments. Error bars represent SDs (*p < 0.05; ***p < 0.005).

Next, we investigated whether, similar to FRAXA (Bjerregaard et al., 2018), FOLD1 is missegregated in mitosis under folate stress conditions. To this end, we treated cells with FdU for 17 h, or deprived them of folate for 3 days, synchronized them in late G2 with the CDK1 inhibitor RO3306, and then released them into mitosis to harvest anaphase/telophase cells (Supplementary Figure 4A). We observed that FOLD1 was unevenly segregated in over 20% of the anaphase cells in response to folate stress but not under control conditions (Supplementary Figures 4B,C). To define whether this missegregation was in some way selective for FOLD1 or whether instead could affect the entire Chr2, we used a previously described FISH-based protocol to examine the fate of two regions on Chr2: FOLD1 and a region on the long arm of Chr2 (henceforth referred as Chr2Q), in cytokinesis-blocked binucleated cells (pseudo-G1 cells) (Figure 3A and Supplementary Figure 1A). We observed a fivefold increase in the frequency of nondisjunction of both FOLD1 and Chr2Q, indicative of whole Chr2 missegregation within one cell cycle of FdU treatment (Figures 3B,C,E). To evaluate the frequency of nondisjunction in other genomic regions that do not contain CG-rich TNRs, we conducted the same analysis with a FISH probe targeting the Chr1CCEN region. Our data indicated that the segregation of Chr1CCEN is not significantly affected by FdU treatment (Figures 3D,E). Intriguingly, and in contrast to what we observed previously through the analysis of FRAXA (Bjerregaard et al., 2018), FOLD1 did not localize to DNA bridges or laggards in anaphase cells (Supplementary Figure 4), or to micronuclei in the pseudo-G1 cells following the folate stress treatment (Figure 3).

### Long-Term Folate Deprivation Causes Chr2 Aneuploidy

Our previous study reported severe FRAXA and ChrX instability in response to prolonged folate deprivation (Bjerregaard et al., 2018). To investigate whether this was also the case for FOLD1 and Chr2, we cultured the cells in the absence of folate for 5 days and analyzed the segregation of FOLD1 using FISH probes targeting FOLD1 or Chr1CCEN in anaphase cells (Supplementary Figure 5A). We observed that FOLD1 was either lost or missegregated in over 25% of the cells, while Chr1CCEN was segregated normally (Supplementary Figures 5B,C). Intriguingly, we could not observe FOLD1 located in between the two separating daughter DNA masses, indicating that the missegregation of FOLD1 was caused by the nondisjunction of two sister chromatids. Nonetheless, these findings are consistent with the observed missegregation of FOLD1 in cells treated with FdU or cultured in the absence of folate for 3 days (Supplementary Figures 4B,C).

**FIGURE 4:**
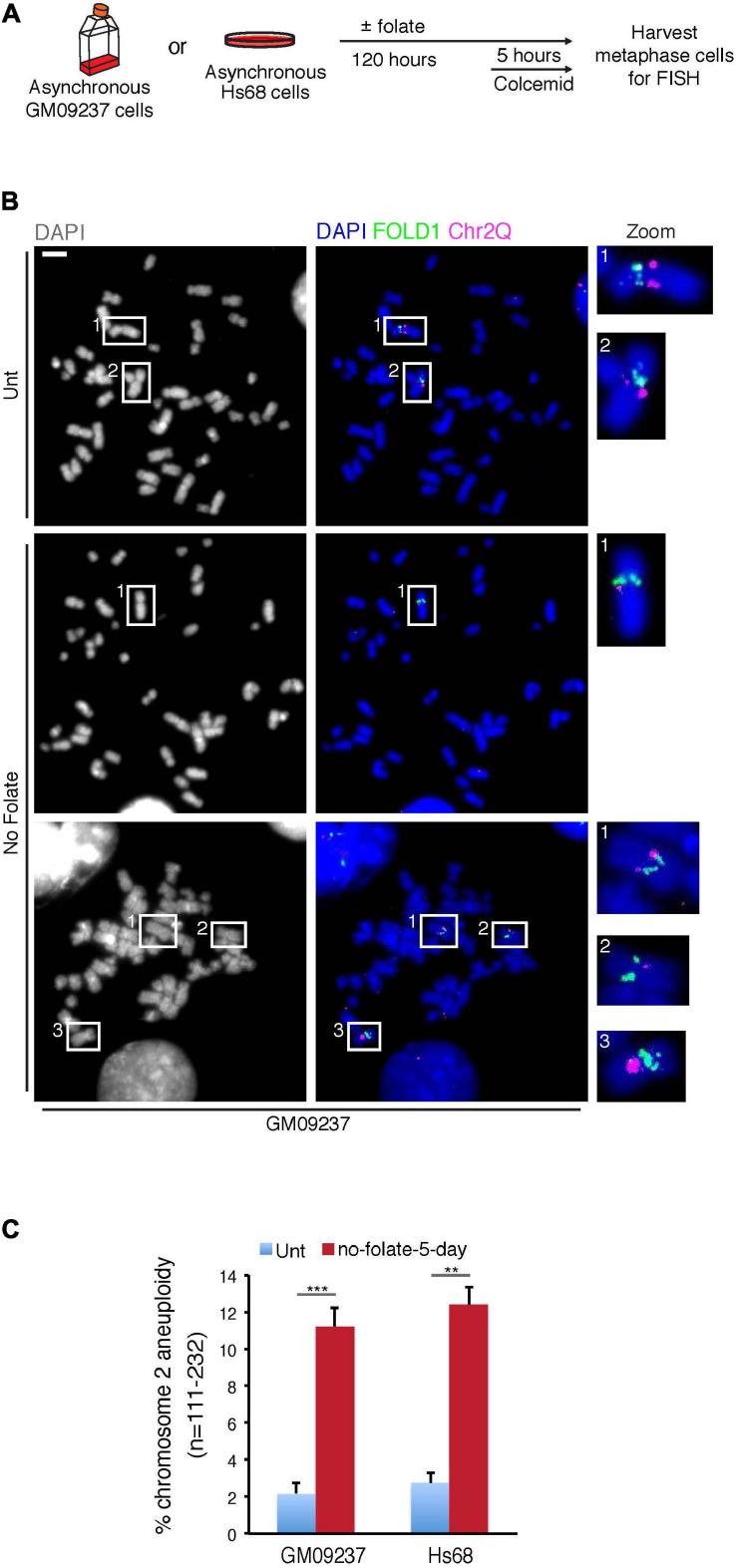
Extended folate deprivation causes Chr2 aneuploidy. **(A)** Experimental workflow for the analysis of metaphase chromosomes following folate deprivation for 5 days. **(B)** Representative images and **(C)** quantification of chromosome 2 aneuploidy in GM09237 and Hs68 cells. Selected regions (white boxes) are shown as zoomed images on the right. *N*: number of metaphase spreads analyzed. Scale bar, 5 μm. Data are means of at least three independent experiments. Error bars represent SDs. (**p < 0.01; ***p < 0.005).

To define more specifically whether the abnormal segregation of FOLD1 was associated with whole or partial Chr2 missegregation, we analyzed metaphase chromosome preparations from cells that had been cultured for 5 days in the absence of folate (Supplementary Figure 5D and Figure 4A). Using FISH probes targeting FOLD1, Chr2Q or Chr1CCEN in the metaphase spread analysis, we observed that FOLD1 and Chr2Q were frequently lost or gained (Figures 4B,C), while the Chr1CCEN region did not show an increased frequency of copy number changes upon prolonged folate deprivation (Supplementary Figures 5E,F). Taken together, these results demonstrate that long-term folate deficiency has a specific effect on the frequency of Chr2 aneuploidy (Figure 4).

### Sequence Analysis of the FOLD1 Region

To verify the sequence of FOLD1 region, we attempted to use PCR to amplify and then sequence this region using the Sanger method. However, after numerous attempts, we could not obtain reasonable PCR products to pursue Sanger sequencing. We therefore performed shot-gun WGS and long-range PacBio sequencing of DNA samples extracted from GM09237 cells cultured with either normal medium (untreated) or medium with no folate for 5 days (no-folate-5-day).

**FIGURE 5:**
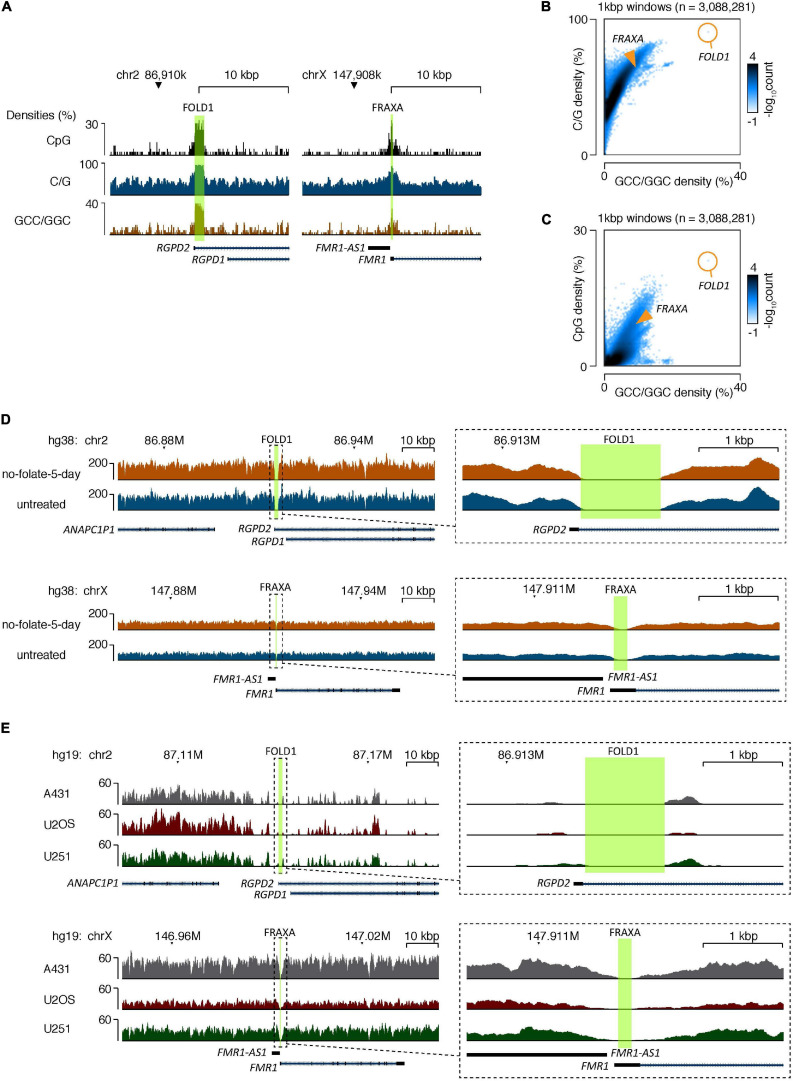
The CG-rich repeats at FOLD1 or FRAXA regions are difficult to be sequenced by second generation of sequencing due to high C/G content. **(A)** Genome browser tracks showing the densities of CpG dinucleotides (top), G/C mononucleotides (middle), and GCC/GGC trinucleotides (bottom) at the FOLD1 (left) and FRAXA (right) regions. The hg38 reference database was used. **(B,C)** 2D-histograms showing the genome-wide relationship between densities of G/C mononucleotides **(B)** or CpG dinucleotides **(C)** on *Y*-axes and GCC/GGC trinucleotides on *X*-axes. Genomic sequences were analyzed in 1 kbp windows, and the number of windows with a particular combination are illustrated by the coloring. The composition of the FRAXA and FOLD1 regions is illustrated with orange overlays. The hg38 reference database was used. **(D)** Genome browser tracks showing the densities of mapped reads from whole-genome sequencing (WGS) of GM09237 cells treated with no folate for 5 days (orange) and untreated cells (blue) at the FOLD1 (top) and FRAXA (bottom) regions. The areas covered by the regions are highlighted in green, and the right-side inserts show zoomed tracks of the regions and their immediate surroundings. The hg38 genome database was used as reference. **(E)** Genome browser tracks showing the densities of mapped reads at the FOLD1 (top) and FRAXA (bottom) regions based on published WGS of A431, U2OS, and U251 cell lines. Data processing and mapping setting was preserved from the published data (Akan et al., 2012). The hg19 genome database was used as reference. The areas covered by the regions analyzed are highlighted in green. The right-side inserts show zoomed tracks of the regions and their immediate surroundings.

The data generated from shot-gun WGS demonstrated that, first, it was not possible to locate repeat rich sequences at the central part of the FOLD1 region in either of the samples, despite the fact we could successfully map the “pseudoreads” generated from the reference genome at this region using our algorithm settings (Figures 5A–C). We reasoned that this is because of the high CG content of the sequences of this region, which could inhibit the amplification and sequencing reactions during the sequencing process. We have also analyzed the FRAXA locus in the same fashion. We found that it is impossible to locate the CGG repeat sequences originating from the central part of the FRAXA region in the two samples either (Figures 5A–C). Second, the sequences of the reads that did map to the region and possible unmapped overhangs were very similar in the untreated and no-folate samples (Figure 5D). Therefore, there was no indication of obvious deletions or insertions surrounding the FOLD1 region in the “no-folate-5-day” samples, which is consistent with our data of MiDAS not occurring at FOLD1 (Supplementary Figure 3). Furthermore, we investigated whether the sequencing data mapped to these regions in our study were equal to those obtained in other WGS data. For this, we analyzed published WGS data of U2OS, A431, and U251MG cells (Akan et al., 2012) in the same way as above. Our analysis confirmed that, indeed, standard shot-gun WGS could not provide the detailed sequences in the FOLD1 or FRAXA regions in these cell lines either (Figure 5E).

The data generated by PacBio sequencing corresponds to 7× or 4× coverage for untreated or no-folate-5-day samples respectively (using hg38 as the reference genome). Because of the low coverage read, we could only locate 10 reads partially or fully covering the FOLD1 region, amongst which 4 reads cover the whole FOLD1 region with 60–82% match (Supplementary Table 3 and Supplementary Figure 6). In general, the reads from either untreated or no-folate-5-day samples contained various mismatched sequences, which were single nucleotide changes, small deletions or insertions. It is well-known that PacBio Sequel sequencing has frequent read errors. This is usually mitigated by making consensus sequences from a substantial number of reads, which cannot be achieved in our case due to the low magnitude of sequencing. Therefore, we could not validate whether the mismatches in the Pacbio reads mapped to FOLD1 were due to sequencing errors, or the actual mutations caused by folate deficiency. In the meantime, as a control, we could identify four reads mapped to the FRAXA region, with one read covering the whole the FRAXA repeat locus (Supplementary Table 3 and Supplementary Figure 6). Our data indicated that the FRAXA locus in GM09237 cells has 931 CGG repeats, which is consistent with previous reports based on Southern blot analysis or allele specific PCRs (Vincent et al., 1991; Seneca et al., 2012).

We conclude that it is very difficult to PCR amplify or obtain the sequences of regions harboring long CG rich repeats (e.g., FOLD1 or FRAXA) using shot-gun WGS. Instead, deep, long-range sequencing (such as Pacbio) is required to verify the sequences at these regions. In addition, based on our Pacbio sequencing data and the Chr2 analysis via FISH (Figure 2), where a portion of cells have two “bent” Chr2s upon folate stress, we propose that GM09237 cell line has homozygous FOLD1 sequence.

## Discussion

Based on the previous knowledge of fragility and missegregation at the abnormally expanded FRAXA locus under folate stress (Santoro et al., 2012; Bjerregaard et al., 2018; Garribba et al., 2020), we investigated whether folate deficiency could perturb other genomic regions containing CG-rich repeat sequences. To address this, we analyzed the stability of the FOLD1 region on Chr2, which contains the largest cluster of CG-rich TNRs present in the human genome under folate stress conditions. By analyzing metaphase chromosomes, we observed a significant increase in the frequency of “bending” or “kinking” (but not “gap” or “break”) of Chr2 at the FOLD1 region in response to folate deprivation. To our knowledge, this phenotype has not been described previously in the human genome. Importantly, this effect was not due to the large size of Chr2, as this phenomenon was not observed at a region close to the centromere of Chr1, the largest human chromosome. We could also confirm this “bending Chr2” phenotype in two other human cell lines (Hs68 and U2OS).

Moreover, the “bending” phenomenon was most frequently observed at FOLD1 under conditions of folate stress, but very rarely in untreated cells or cells exposed to other replication-stress-inducing agents such as APH or HU. Thus, the bending at FOLD1 appears to be induced specifically by folate stress. Taking into the account the knowledge gained from previous studies on CFSs and FRAXA, we propose that this increased frequency of “bending” of Chr2 at FOLD1 is likely to be driven by the under-condensation of the DNA due to delayed replication at this region under folate stress condition. It is possible that, because CG-rich TNRs tend to form DNA secondary structures (Fry and Loeb, 1994; Usdin and Woodford, 1995) and assemble into nucleosomes with low efficiency after replication (Wang et al., 1996), folate stress exacerbates the replication-related difficulties of the FOLD1 region, thus causing its delayed condensation in early mitosis under this condition. Nevertheless, we could not rule out the possibility that folate deficiency could cause changes in replication timing and transcription of genes located in the FOLD1 region (e.g., the *RGPD2* gene), which could then contribute to the delayed condensation at this region. Future work is needed to validate these possibilities.

Interestingly, our data also suggested that FOLD1 differs from FRAXA in that it is fully replicated when cells enter mitosis. This is based on the following observations: (1) FOLD1 does not localize to DNA bridges/laggards in anaphase, or to micronuclei in the newly-born G1 cells; (2) MiDAS is apparently absent from bent FOLD1; and 3) both sister chromatids look very similar at bent FOLD1, whereas the expanded FRAXA locus generally shows the conventional appearance of fragility, with a break/gap on a single sister chromatid only (Bjerregaard et al., 2018). To explain this apparent difference, it is important to note that the CG-rich TNRs at FOLD1, despite being over 900 nucleotides in length, are interrupted by A/T base pairs, with the longest continuous CGG repeat cluster comprising only 17 repeat units (Supplementary Text 1). It was shown previously that interruptions in the TNR sequence suppress repeat instability (Yrigollen et al., 2014), and more than 100 continuous CGG repeats are generally required to cause replication fork blockade (Voineagu et al., 2009). It is likely, therefore, that the CG-rich repeats at FOLD1 lead only to a temporary replication fork perturbation, but not an extended fork blockade. In agreement with this, in response to FdU or 3-day folate deprivation, although FOLD1 was frequently missegregated, we could always observe four copies of this region (in total) distributed between the two daughter cells (Supplementary Figure 4B and Figure 3B), which was not the case for FRAXA (Bjerregaard et al., 2018). Taken together, we propose that the bending (or “kink”) at FOLD1 simply reflects the perturbed condensation of a region due to delayed replication in interphase under folate stress conditions. In comparison with FRAXA, which appears fragile in metaphase and localizes on DNA bridges in anaphase upon folate stress (Bjerregaard et al., 2018), FOLD1 displays a milder phenotype in this respect.

Despite the fact that FOLD1 appears less susceptible to folate stress than the expanded FRAXA locus, we observed marked missegregation of the FOLD1 region, as well as the whole Chr2. In this regard, it might be significant that FOLD1 is adjacent to the Chr2 centromere, which might influence its mobility in mitosis. For example, as discussed above, the delayed condensation of FOLD1 might influence accurate microtubule attachment or the release of cohesin from Chr2 centromeres, which would then lead to nondisjunction of sister chromatids of this chromosome. A more direct effect of FOLD1 is also possible, based on the likelihood that unfinished replication of this locus might precipitate a failure to decatenate the two Chr2 sister-chromatids in a timely fashion, leading to Chr2 non-disjunction. Due to limited type and number of cells analyzed, it remains to be clarified whether the frequencies of missegregation of FOLD1 region or the whole Chr2 is the same in a larger collection of different cell types. Nonetheless, in line with our data on FOLD1’s copy number changes, it was observed that there is an increase of copy number of Chr2p11.2 in several tumor types (Mastracci et al., 2006; Oh et al., 2012). In addition, Chr2p11.2 is also the location of frequent deletions or insertions, some of which, particularly large deletions, are associated with human diseases (Firth et al., 2009) (Supplementary Tables 4, 5). Judging by the data from this study, we reckon this would most likely be caused by the mistakes occurring in S phase when this region is being replicated in the cells. Future studies are warranted to analyze the cellular functions of the genes located in this region.

In summary, we have demonstrated that nondisjunction of Chr2 is promoted by folate deficiency via a CG-rich TNR region (Supplementary Figure 7). Future studies are warranted to identify the proteins or pathways underlying this missegregation, and how this could lead to the altered expression of the genes located at this region. Considering that CG-rich TNRs are significantly enriched at genomic regions associated with the regulation of gene expression, our findings provide a new line of evidence to explain why folate deficiency is associated with a wide range of human disorders including cancers. In addition, because such chromosomal changes induced by folate deficiency could potentially be irreversible, our results highlight the importance of maintaining optimal folate levels irrespective of age or gender in human population.

## Data Availability Statement

The datasets presented in the study are deposited in the European Genome-phenome Archive (EGA) repository, accession number (EGAD00001007732).

## Author Contributions

LG, MMGD, and LR performed the experiments and data analysis. IV and ML performed the bioinformatic analysis. LG and YL designed the experiments and interpreted results. LG, IV, ML, and YL wrote the manuscript. All authors edited it.

## Conflict of Interest

The authors declare that the research was conducted in the absence of any commercial or financial relationships that could be construed as a potential conflict of interest.

## Footnotes

1 https://www.ncbi.nlm.nih.gov/assembly/GCF_000001405.26/

## Supplementary Material

Supplementary Figure 1 (Related to Figures 2–4) The background information of this study. **(A)** A diagram depicting the genomic location of the BAC clones used in FISH analyses. **(B)** Experimental workflow for the analysis of FRAXA fragility upon FdU treatment on metaphase spreads of GM09237 cells. **(C)** Representative images of the FRAXA locus (green) on metaphase chromosomes in GM09237 cells. The yellow arrow indicates an example of a fragile FRAXA locus. **(D)** Quantification of fragility at FRAXA following FdU treatment. *N*: number of ChrX analyzed. Scale bar, 5 μm. Error bars represent SDs from at least three experiments. Error bars represent SD. (^****^*p* < 0.001).

Supplementary Figure 2 (Related to Figure 2) Chr2 exhibits bending at FOLD1 in Hs68 and U2OS cell lines upon folate stress. **(A)** Experimental workflow for the analysis of metaphase cells by FISH or karyotyping following FdU treatment for 22 h (FdU) or folate deprivation for 3 days (No folate) in GM09237, Hs68, or U2OS. U2OS cells were not included in karyotyping analysis since it is an aneuploid cell line. **(B)** Quantification of Chr2 or Chr1 bending at either FOLD1 or Chr1CCEN, respectively in Hs68 and U2OS cell lines under “FdU” or “No folate” conditions. *N*: number of Chr2s or Chr1s identified and analyzed by FISH probes targeting FOLD1 or Chr1CCEN respectively. **(C)** Representative karyotype and **(D)** quantification of Chr2 bending at the Chr2p11.2 that contains FOLD1 region (arrowed) in GM09237 and Hs68 cells. *N*: number of Chr2s analyzed in karyotype analysis. Chr1 and Chr3 were analyzed in parallel. Error bars represent SDs from at least three experiments (ns, not significant; ^∗^*p* < 0.05; ^∗∗^*p* < 0.01; ^****^*p* < 0.001).

Supplementary Figure 3 Mitotic DNA synthesis does not occur at FOLD1 following FdU treatment. **(A)** Experimental workflow for the analysis of EdU incorporation at FOLD1 in mitosis upon FdU treatment in GM09237 cells. **(B)** Representative images of bent FOLD1 (green) negative for EdU incorporation (magenta). The FRAXA locus was used as a positive control. Scale bar, 5 μm.

Supplementary Figure 4 (Related to Figure 3) FOLD1 missegregation in anaphase following FdU treatment or folate deprivation. **(A)** Experimental workflow for the analysis of missegregation in anaphase cells following FdU treatment for 17 h (FdU) or folate deprivation for 3 days (No folate) in GM09237 cells. **(B)** Representative images and **(C)** quantification of FOLD1 missegregation in anaphase GM09237 cells. White arrowheads indicate the location of FOLD1. *N*: number of anaphase cells analyzed. Scale bar, 5 μm. Data are means of at least three independent experiments. Error bars represent SD (ns, not significant).

Supplementary Figure 5 (Related to Figure 4) Long-term effects of folate deprivation in anaphase cells and metaphase spreads. **(A)** Experimental workflow for analysis of anaphase cells following folate deprivation for 5 days. **(B)** Representative images and **(C)** quantification of missegregation/loss of FOLD1 in GM09237 cells. The Chr1CCEN region was used as a control. White arrowheads indicate the location of FOLD1. *N*: number of anaphase cells analyzed. **(D)** Experimental workflow for the analysis of metaphase chromosomes following folate deprivation for 5 days. **(E)** Representative images and **(F)** quantification of copy number changes of FOLD1 in metaphase chromosomes in GM09237 cells. The Chr1CCEN region was used as a control. Selected regions (numbered white boxes) are shown below as zoomed images. *N*: number of metaphase spreads analyzed. Scale bar, 5 μm. Data are means of at least three independent experiments. Error bars represent SDs (^∗^*p* < 0.05; ^∗∗∗^ *p* < 0.005; ^****^*p* < 0.001).

Supplementary Figure 6 (Related to Figure 5) Examples of PacBio reads containing sequences of FOLD1 or FRAXA obtained from GM09237 cells. **(A)** A diagram of the alignment of a PacBio sequencing read (m54282_190714_174122/27394352/7413_34584), a part of Chr2 (hs38) and a FOLD1 sequence (hg38). The sequence of read m54282_190714_174122/27394352/7413_34584 matching to FOLD1 is marked in purple and shown in the lower panel. **(B)** A diagram of the alignment of a PacBio sequencing read (m54281_190714_174033/27198327/934_17319) and the sequences of 5′ or 3′ side of the FRAXA CGG repeats located in FMR1 exon 1 (hs38). The abnormally expanded 900 CGG repeats in the m54281_190714_174033/27198327/934_17319 read is marked in purple and shown in the lower panel. The alignment diagrams were created by Sequencher program.

Supplementary Figure 7 A graphic summary of the key findings. When cells are grown under folate stress conditions, replication at FOLD1 is delayed, but is nevertheless completed in interphase. This, however, causes a delay in the condensation at FOLD1 in early mitosis, leading to the “bent” shape of Chr2 with the inflection point at the position of the FOLD1 region. During anaphase, the uncondensed FOLD1 region negatively influences the accuracy of sister chromatid separation, possibly due to its close association with the Chr2 centromere. This leads to FOLD1 and Chr2 nondisjunction in the newly born G1 cells. Because the copy number changes of Chr2 will likely not immediately affect cell growth, several rounds of cell proliferation under folate stress conditions can occur, leading to an increasing number of cells displaying Chr2 aneuploidy.

Supplementary Table 1 (Related to Figure 1) An excel file of the genomic information of all of the AT-, or CG- rich repeats (period ranging from 1 to 14 bp) in human genome database, and the genomic information of AT-, or CG- rich trinucleotide repeats that have more than 50 copies in human genome database. This file contains four data containing sheets. The headings for these sheets are: “AT-rich_with_features,” “GC-rich_with_features,” “AT-rich_period3_copynumber > 50,” and “GC-rich_period3_copynumber > 50.” The categories of the information included are: the location of the repeat (chromosome number, chromStart – chromEnd, cytogenetic Band of the repeat, cytoBand period length, copy Numbers of the repeat, total Length of the repeat, perfect Match percentage, sequence of the repeat region, database source), and the overlap with functional features of the genome as described in Figure 1. The link to access this file is at: https://drive.google.com/open?id=1Mk3oFO4dxUNEosf-4pPPmJfzbugEi2Tl.

Supplementary Table 2 (Related to Figure 1B) The data table for the plots shown in Figure 1B.

Supplementary Table 3 (Related to Figure 5) The sequencing reads generated by Pacbio sequencing containing partial or full FOLD1 or FRAXA regions. This file contains 2 data containing sheets. The headings for these sheets are: “Positive_PacBio_reads_for_FOLD1” and “Positive_PacBio_reads_for_FRAXA.”

Supplementary Table 4 A summary of the polymorphisms at FOLD1 (chr2:86913957-86914954) present in the general population.

Supplementary Table 5 A summary of the mutations in patient reported in DECIPHER database containing FOLD1 (chr2:86913957-86914954) (DECIPHER: https://decipher.sanger.ac.uk/).

Supplementary Text 1 Sequence of the FOLD1 CG-rich trinucleotide region (chr2:86913957-86914954; hg38). A/T interruptions are highlighted in bold.
