# Supplementary figures and images for: Folate Deficiency Triggers the Abnormal Segregation of a Region With Large Cluster of CG-Rich Trinucleotide Repeats on Human Chromosome 2
Source: Front Genet. 2021 Jul 1;12:695124. doi: 10.3389/fgene.2021.695124 (PMC8281231; doi:10.3389/fgene.2021.695124)

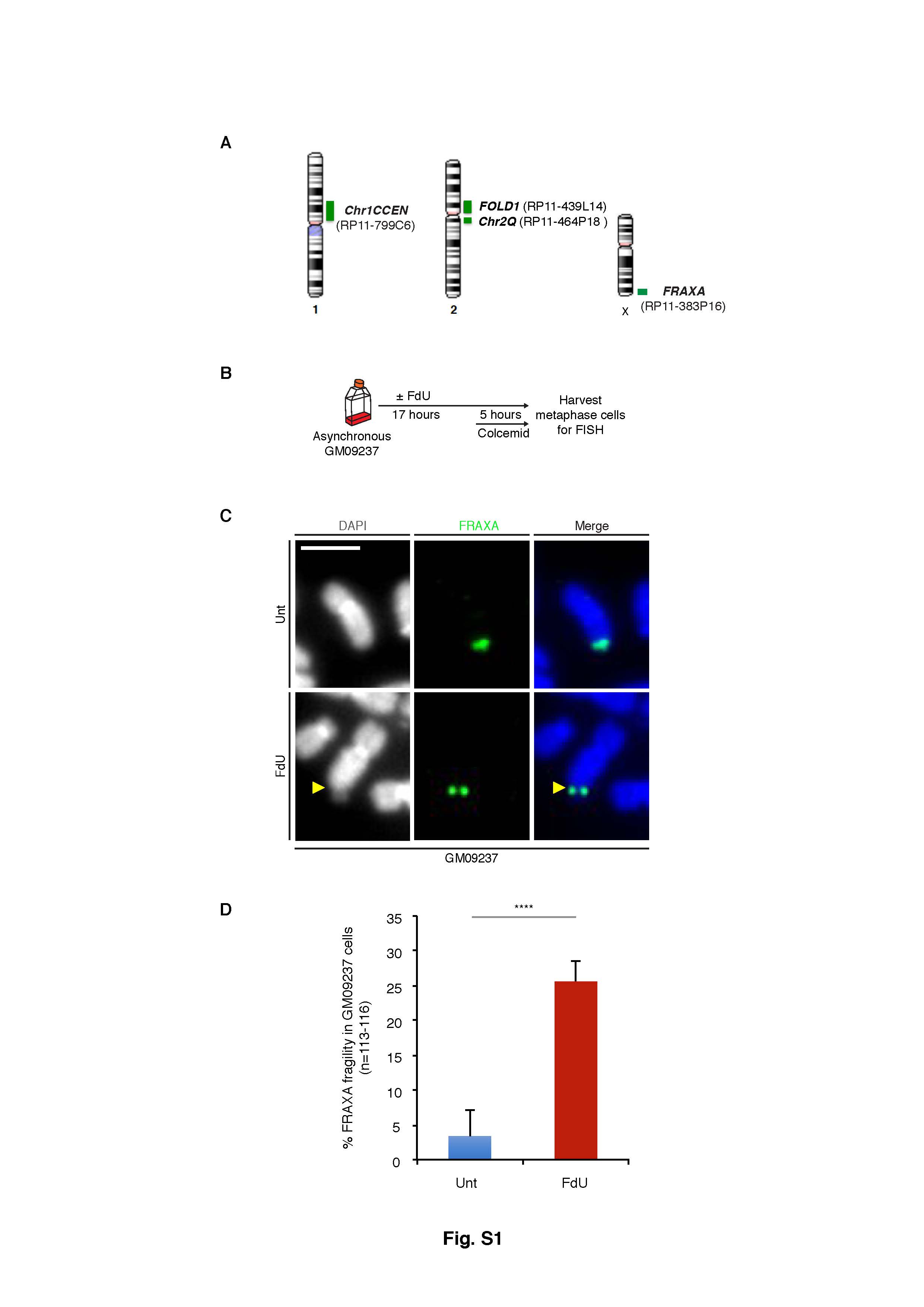

Supplement: Supplementary Figure 1 — (Related to Figures 2–4) The background information of this study. (A) A diagram depicting the genomic location of the BAC clones used in FISH analyses. (B) Experimental workflow for the analysis of FRAXA fragility upon FdU treatment on metaphase spreads of GM09237 cells. (C) Representative images of the FRAXA locus (green) on metaphase chromosomes in GM09237 cells. The yellow arrow indicates an example of a fragile FRAXA locus. (D) Quantification of fragility at FRAXA following FdU treatment. N: number of ChrX analyzed. Scale bar, 5 μm. Error bars represent SDs from at least three experiments. Error bars represent SD. (****p < 0.001). [file Image_1.JPEG]

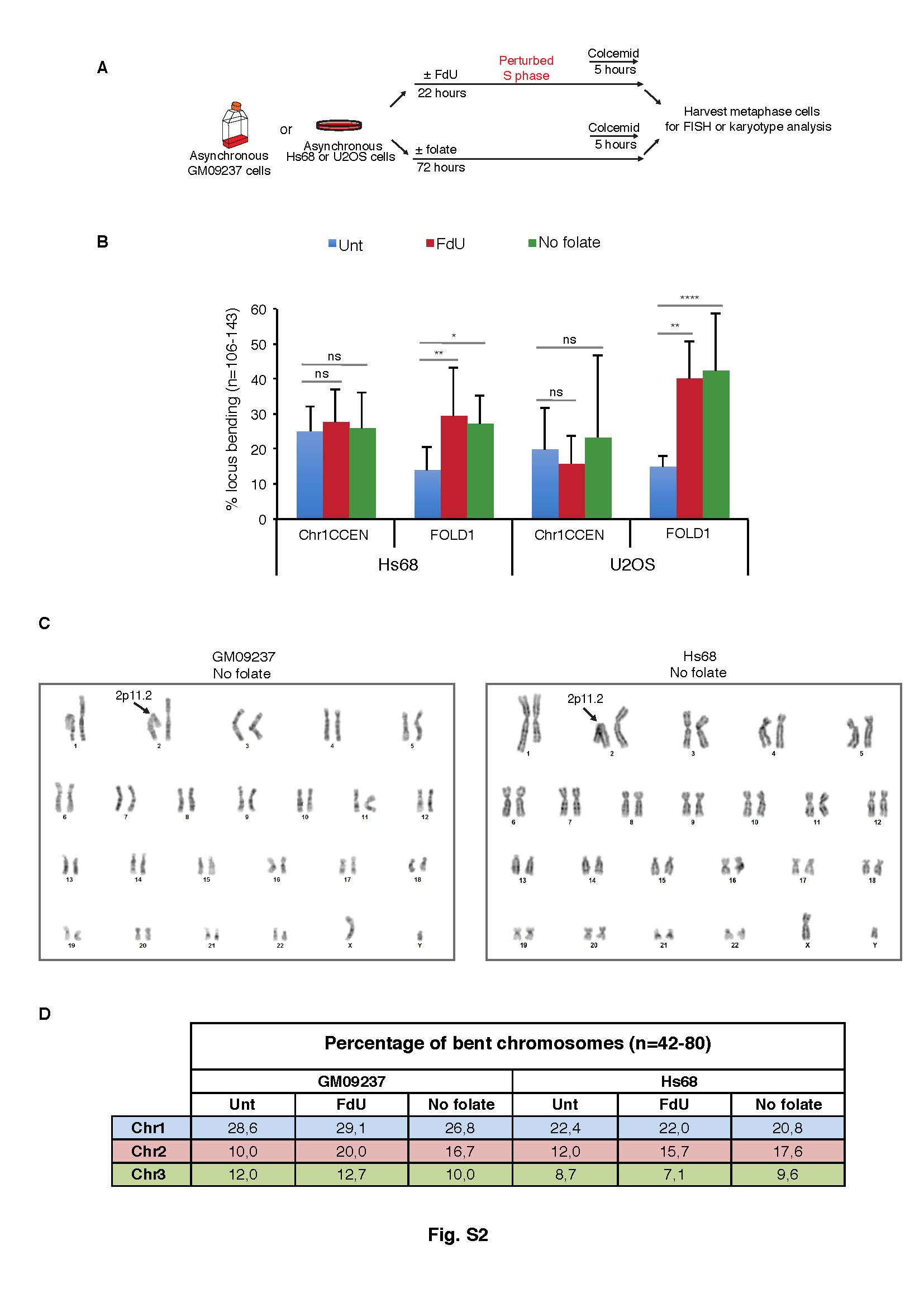

Supplement: Supplementary Figure 2 — (Related to Figure 2) Chr2 exhibits bending at FOLD1 in Hs68 and U2OS cell lines upon folate stress. (A) Experimental workflow for the analysis of metaphase cells by FISH or karyotyping following FdU treatment for 22 h (FdU) or folate deprivation for 3 days (No folate) in GM09237, Hs68, or U2OS. U2OS cells were not included in karyotyping analysis since it is an aneuploid cell line. (B) Quantification of Chr2 or Chr1 bending at either FOLD1 or Chr1CCEN, respectively in Hs68 and U2OS cell lines under “FdU” or “No folate” conditions. N: number of Chr2s or Chr1s identified and analyzed by FISH probes targeting FOLD1 or Chr1CCEN respectively. (C) Representative karyotype and (D) quantification of Chr2 bending at the Chr2p11.2 that contains FOLD1 region (arrowed) in GM09237 and Hs68 cells. N: number of Chr2s analyzed in karyotype analysis. Chr1 and Chr3 were analyzed in parallel. Error bars represent SDs from at least three experiments (ns, not significant; ∗p < 0.05; ∗∗p < 0.01; ****p < 0.001). [file Image_2.JPEG]

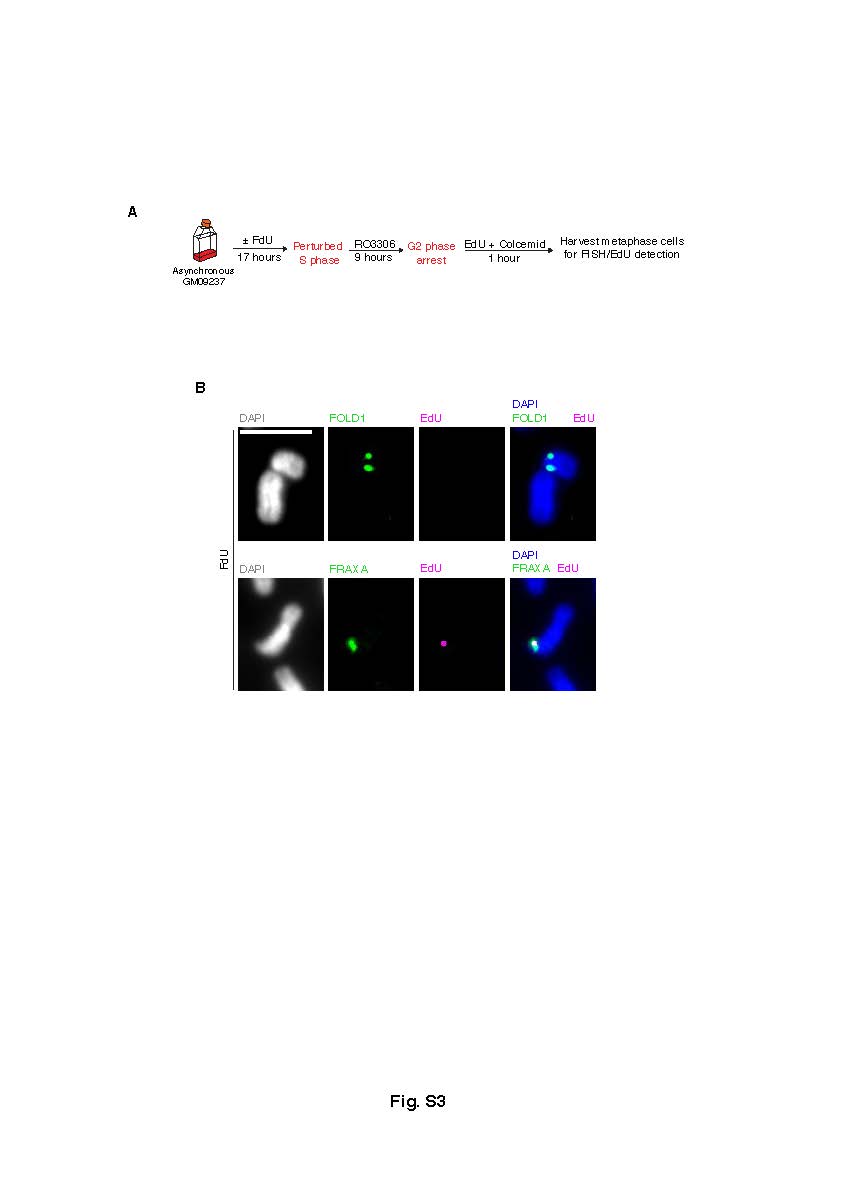

Supplement: Supplementary Figure 3 — Mitotic DNA synthesis does not occur at FOLD1 following FdU treatment. (A) Experimental workflow for the analysis of EdU incorporation at FOLD1 in mitosis upon FdU treatment in GM09237 cells. (B) Representative images of bent FOLD1 (green) negative for EdU incorporation (magenta). The FRAXA locus was used as a positive control. Scale bar, 5 μm. [file Image_3.JPEG]

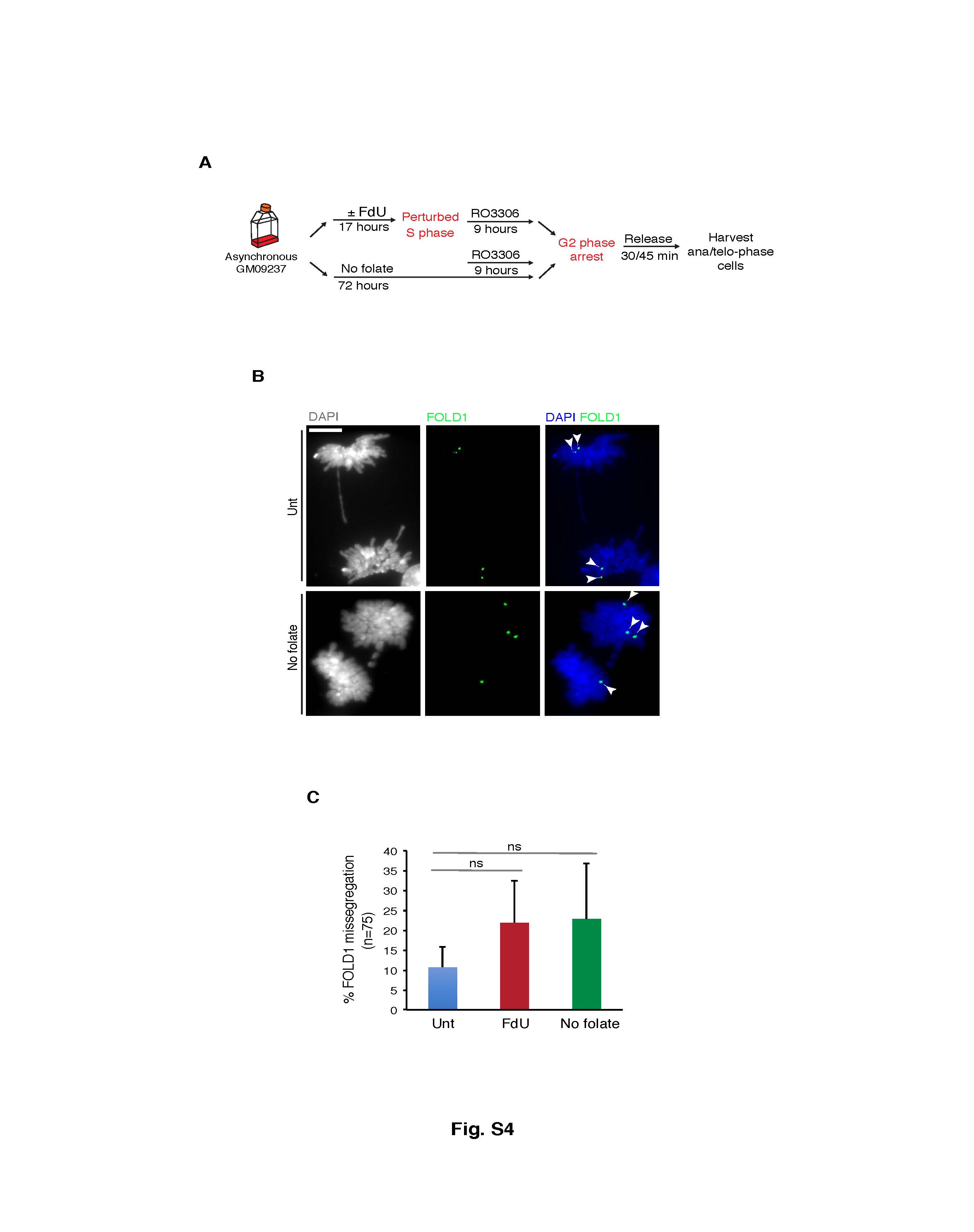

Supplement: Supplementary Figure 4 — (Related to Figure 3) FOLD1 missegregation in anaphase following FdU treatment or folate deprivation. (A) Experimental workflow for the analysis of missegregation in anaphase cells following FdU treatment for 17 h (FdU) or folate deprivation for 3 days (No folate) in GM09237 cells. (B) Representative images and (C) quantification of FOLD1 missegregation in anaphase GM09237 cells. White arrowheads indicate the location of FOLD1. N: number of anaphase cells analyzed. Scale bar, 5 μm. Data are means of at least three independent experiments. Error bars represent SD (ns, not significant). [file Image_4.JPEG]

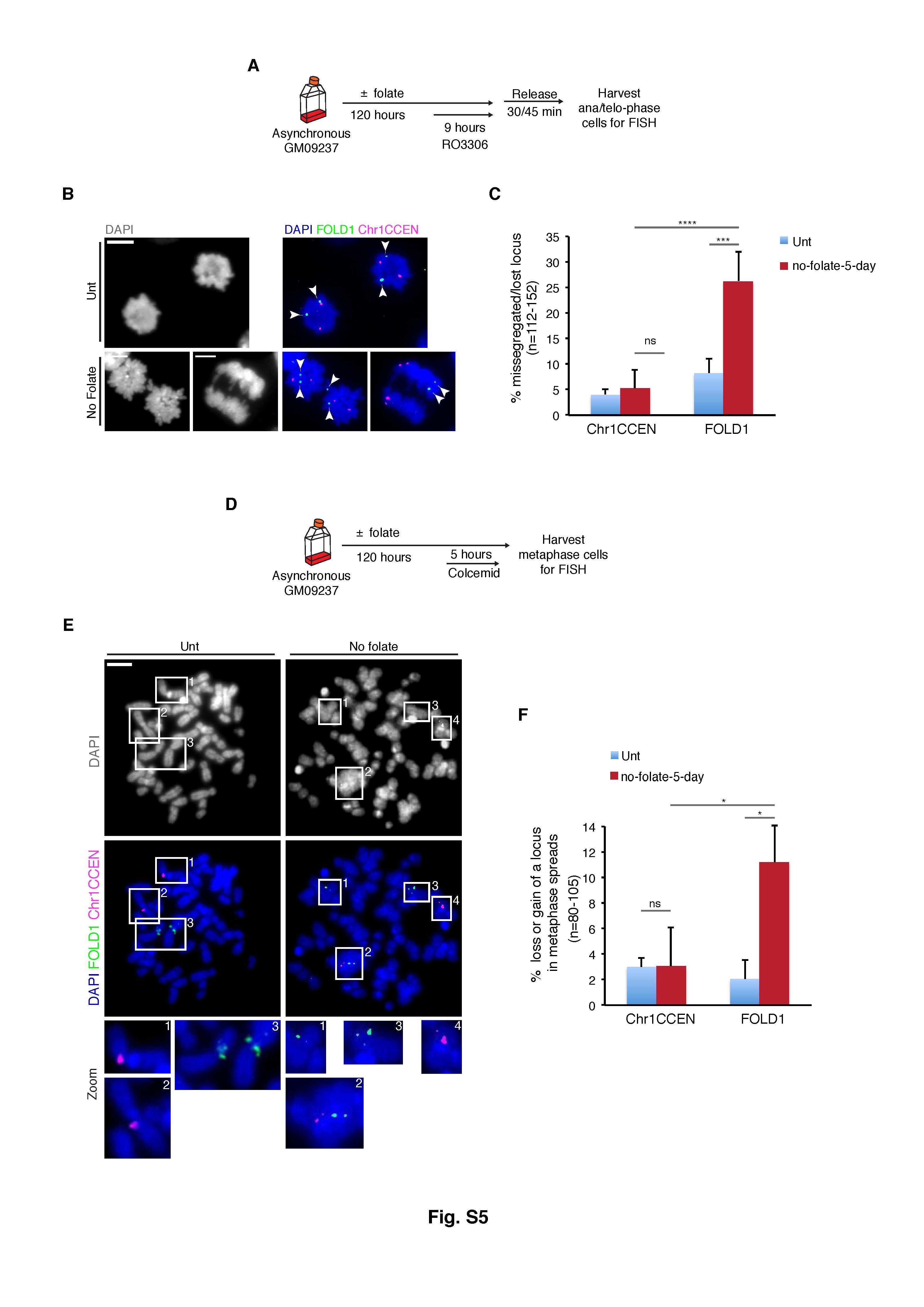

Supplement: Supplementary Figure 5 — (Related to Figure 4) Long-term effects of folate deprivation in anaphase cells and metaphase spreads. (A) Experimental workflow for analysis of anaphase cells following folate deprivation for 5 days. (B) Representative images and (C) quantification of missegregation/loss of FOLD1 in GM09237 cells. The Chr1CCEN region was used as a control. White arrowheads indicate the location of FOLD1. N: number of anaphase cells analyzed. (D) Experimental workflow for the analysis of metaphase chromosomes following folate deprivation for 5 days. (E) Representative images and (F) quantification of copy number changes of FOLD1 in metaphase chromosomes in GM09237 cells. The Chr1CCEN region was used as a control. Selected regions (numbered white boxes) are shown below as zoomed images. N: number of metaphase spreads analyzed. Scale bar, 5 μm. Data are means of at least three independent experiments. Error bars represent SDs (∗p < 0.05; ∗∗∗ p < 0.005; ****p < 0.001). [file Image_5.JPEG]

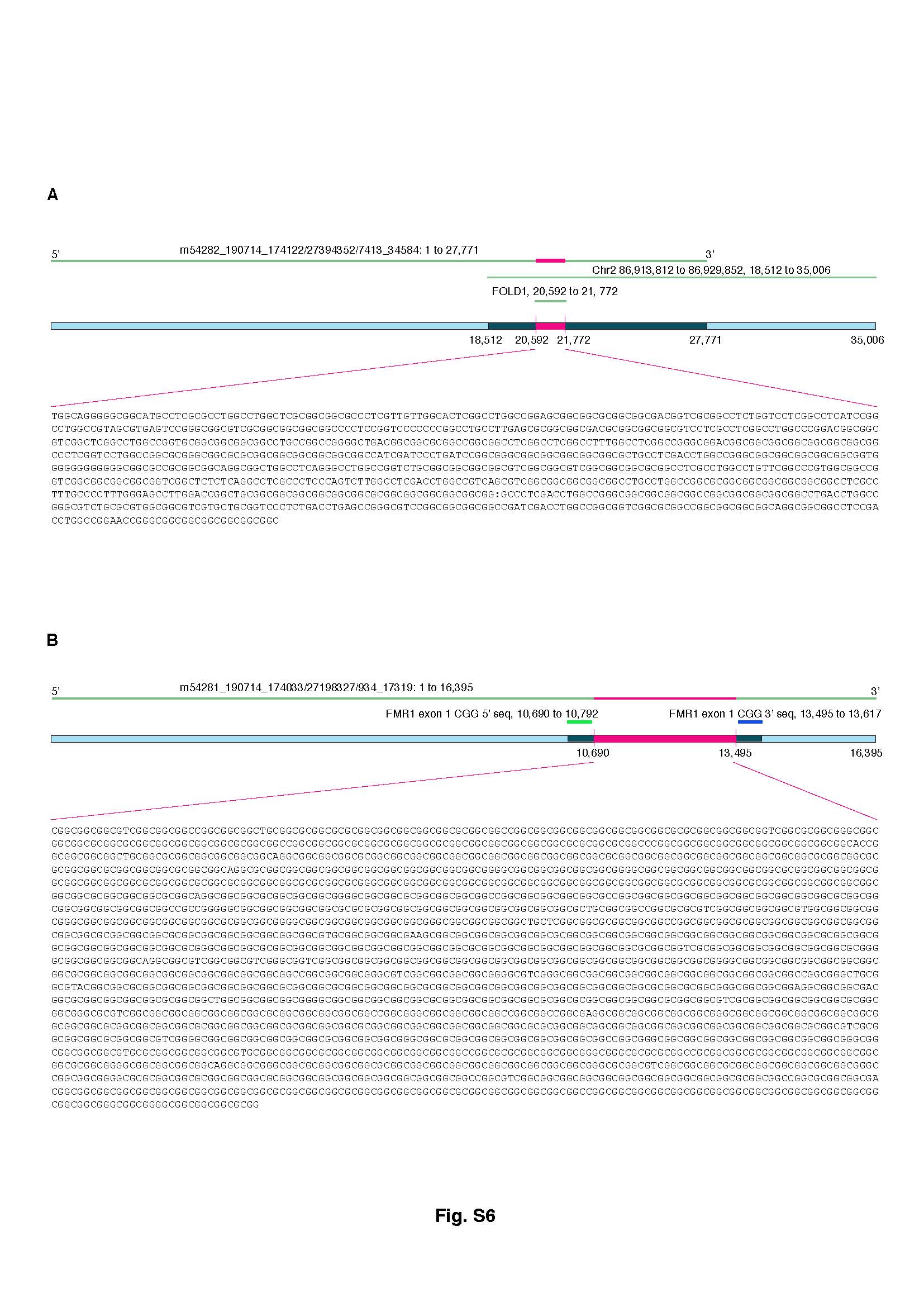

Supplement: Supplementary Figure 6 — (Related to Figure 5) Examples of PacBio reads containing sequences of FOLD1 or FRAXA obtained from GM09237 cells. (A) A diagram of the alignment of a PacBio sequencing read (m54282_190714_174122/27394352/7413_34584), a part of Chr2 (hs38) and a FOLD1 sequence (hg38). The sequence of read m54282_190714_174122/27394352/7413_34584 matching to FOLD1 is marked in purple and shown in the lower panel. (B) A diagram of the alignment of a PacBio sequencing read (m54281_190714_174033/27198327/934_17319) and the sequences of 5′ or 3′ side of the FRAXA CGG repeats located in FMR1 exon 1 (hs38). The abnormally expanded 900 CGG repeats in the m54281_190714_174033/27198327/934_17319 read is marked in purple and shown in the lower panel. The alignment diagrams were created by Sequencher program. [file Image_6.JPEG]

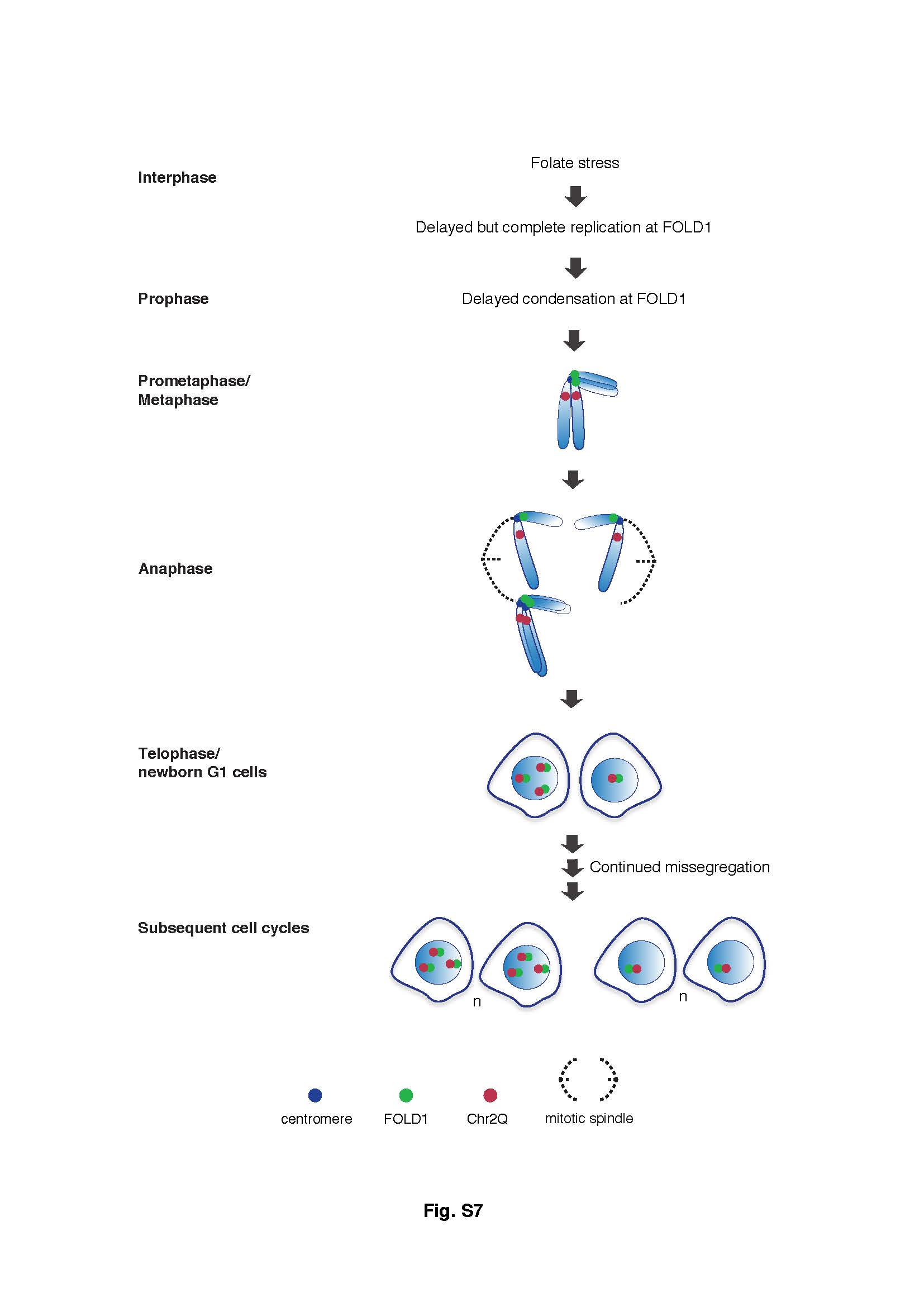

Supplement: Supplementary Figure 7 — A graphic summary of the key findings. When cells are grown under folate stress conditions, replication at FOLD1 is delayed, but is nevertheless completed in interphase. This, however, causes a delay in the condensation at FOLD1 in early mitosis, leading to the “bent” shape of Chr2 with the inflection point at the position of the FOLD1 region. During anaphase, the uncondensed FOLD1 region negatively influences the accuracy of sister chromatid separation, possibly due to its close association with the Chr2 centromere. This leads to FOLD1 and Chr2 nondisjunction in the newly born G1 cells. Because the copy number changes of Chr2 will likely not immediately affect cell growth, several rounds of cell proliferation under folate stress conditions can occur, leading to an increasing number of cells displaying Chr2 aneuploidy. [file Image_7.JPEG]

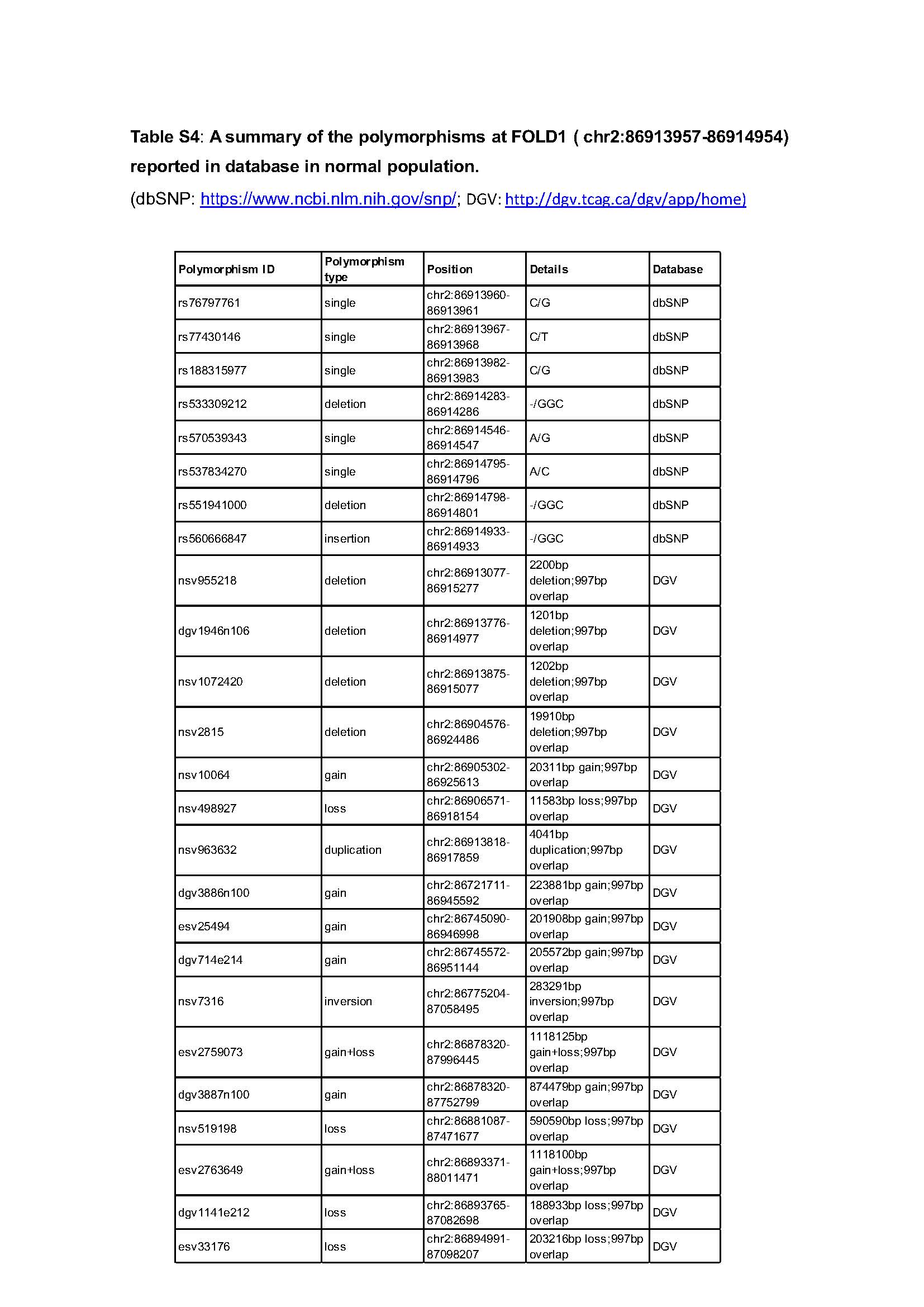

Supplement: Supplementary Table 4 — A summary of the polymorphisms at FOLD1 (chr2:86913957-86914954) present in the general population. [file Image_8.JPEG]

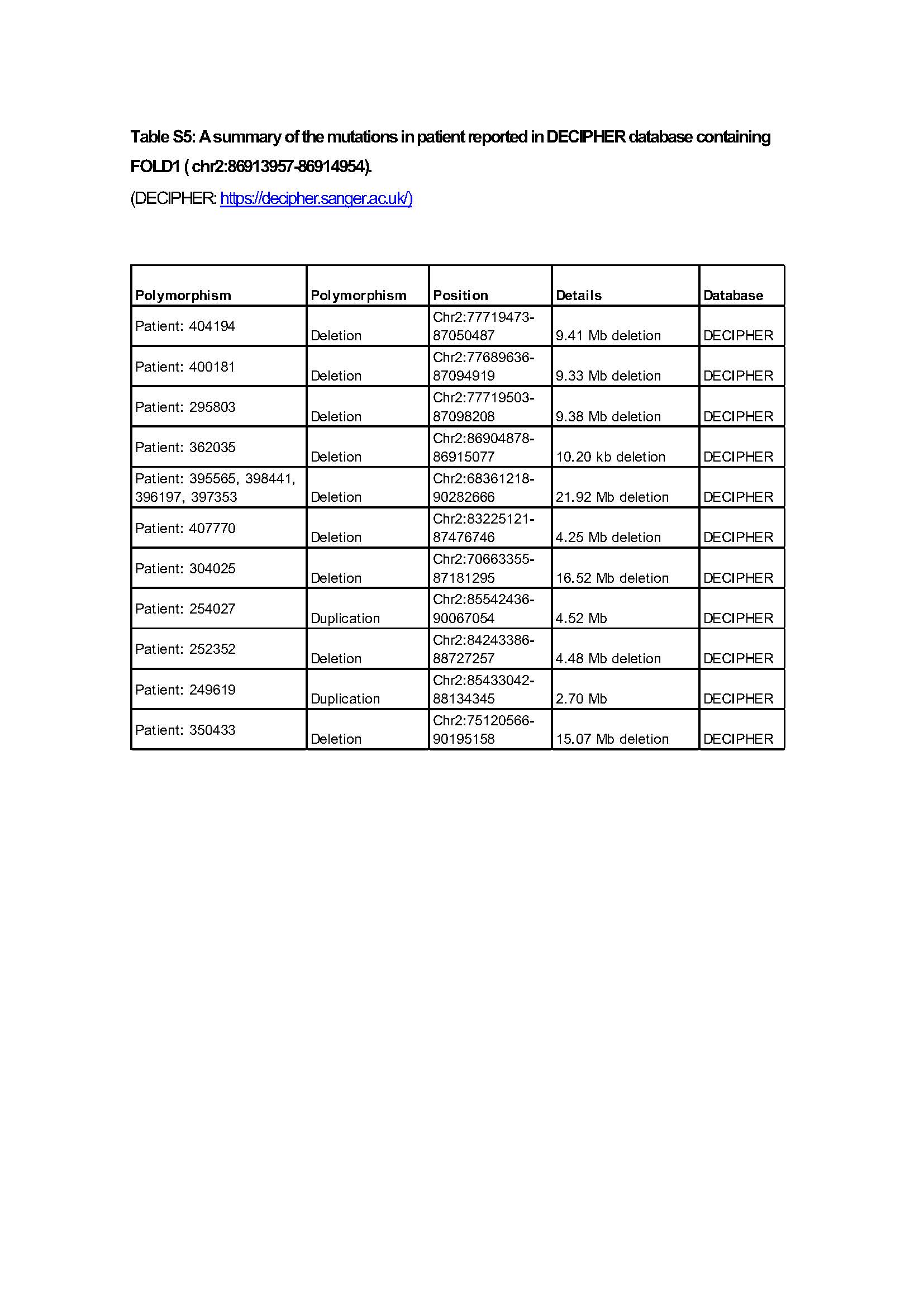

Supplement: Supplementary Table 5 — A summary of the mutations in patient reported in DECIPHER database containing FOLD1 (chr2:86913957-86914954) (DECIPHER: https://decipher.sanger.ac.uk/). [file Image_9.JPEG]

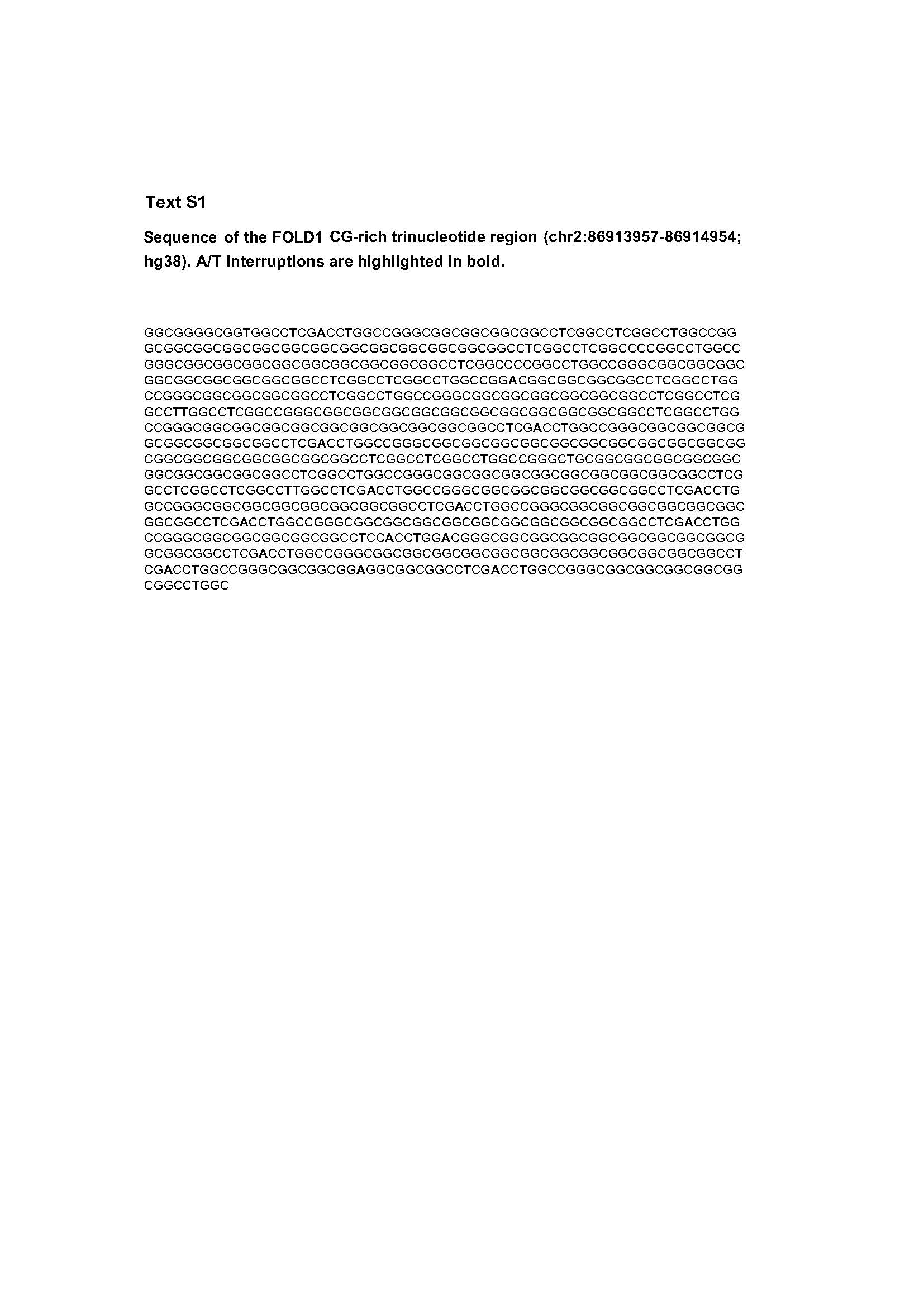

Supplement: Supplementary Text 1 — Sequence of the FOLD1 CG-rich trinucleotide region (chr2:86913957-86914954; hg38). A/T interruptions are highlighted in bold. [file Image_10.JPEG]
