# Supplementary material for: Folate Deficiency Triggers the Abnormal Segregation of a Region With Large Cluster of CG-Rich Trinucleotide Repeats on Human Chromosome 2
Source: Front Genet. 2021 Jul 1;12:695124. doi: 10.3389/fgene.2021.695124 (PMC8281231; doi:10.3389/fgene.2021.695124)
Supplement: Supplementary Table 1 — (Related to Figure 1) An excel file of the genomic information of all of the AT-, or CG- rich repeats (period ranging from 1 to 14 bp) in human genome database, and the genomic information of AT-, or CG- rich trinucleotide repeats that have more than 50 copies in human genome database. This file contains four data containing sheets. The headings for these sheets are: “AT-rich_with_features,” “GC-rich_with_features,” “AT-rich_period3_copynumber > 50,” and “GC-rich_period3_copynumber > 50.” The categories of the information included are: the location of the repeat (chromosome number, chromStart – chromEnd, cytogenetic Band of the repeat, cytoBand period length, copy Numbers of the repeat, total Length of the repeat, perfect Match percentage, sequence of the repeat region, database source), and the overlap with functional features of the genome as described in Figure 1. The link to access this file is at: https://drive.google.com/open?id=1Mk3oFO4dxUNEosf-4pPPmJfzbugEi2Tl. [file Data_Sheet_1.DOCX]

**The Caption and access details for Table S1.**

An excel file of the genomic information of all of the AT-, or CG- rich repeats (period ranging from 1 to 14 bp) in human genome database, and the genomic information of AT-, or CG- rich trinucleotide repeats that have more than 50 copies in human genome database. This file contains 4 data-containing sheets. The headings for these sheets are: ‘AT-rich_with_features’, ‘GC-rich_with_features’, ‘AT-rich_period3_copynumber>50’, and ‘GC-rich_period3_copynumber>50’. The categories of the information included are: the location of the repeat (chromosome number, chromStart – chromEnd, cytogenetic Band of the repeat, cytoBand period length, copy Numbers of the repeat, total Length of the repeat, percent match with the consensus repeat unit, sequence of the repeat region, database source), and the overlap with functional features of the genome as described in Fig. 1.

The link to access this file is at: <https://drive.google.com/open?id=1Mk3oFO4dxUNEosf-4pPPmJfzbugEi2Tl>
